# Repeat Placental Growth Factor-Based Testing in Women With Suspected Preterm Preeclampsia: A Stratified Analysis of the PARROT-2 Trial

**DOI:** 10.1161/HYPERTENSIONAHA.123.22411

**Published:** 2024-05-06

**Authors:** Alice Hurrell, Louise Webster, Jenie Sparkes, Cheryl Battersby, Anna Brockbank, Katherine Clark, Kate E. Duhig, Carolyn Gill, Marcus Green, Rachael M. Hunter, Paul T. Seed, Zoe Vowles, Jenny Myers, Andrew H. Shennan, Lucy C. Chappell

**Affiliations:** Department of Women and Children’s Health, School of Life Course Sciences, King’s College London, United Kingdom (A.H., L.W., J.S., A.B., C.G., P.T.S., K.C., Z.V., A.H.S., L.C.C.).; Division of Developmental Biology and Medicine, Maternal and Fetal Health Research Centre, School of Medical Sciences, Faculty of Biology, Medicine and Health, University of Manchester, Manchester Academic Health Science Centre, United Kingdom (K.E.D., J.M.).; Neonatal Medicine, School of Public Health, Faculty of Medicine, Imperial College London Chelsea and Westminster Hospital Campus, United Kingdom (C.B.).; Action on Pre-Eclampsia, Evesham, United Kingdom (M.G.).; Institute of Epidemiology and Health Care, University College London, United Kingdom (R.M.H.).

**Keywords:** biomarkers, blood pressure, hypertension, pre-eclampsia, pregnancy

## Abstract

**BACKGROUND:**

PlGF (placental growth factor)-based testing reduces severe maternal adverse outcomes. Repeat PlGF-based testing is not associated with improved perinatal or maternal outcomes. This planned secondary analysis aimed to determine whether there is a subgroup of women who benefit from repeat testing.

**METHODS:**

Pregnant individuals with suspected preterm preeclampsia were randomized to repeat revealed PlGF-based testing, compared with usual care where testing was concealed. Perinatal and maternal outcomes were stratified by trial group, by initial PlGF-based test result, and by PlGF-based test type (PlGF or sFlt-1 [soluble fms-like tyrosine kinase-1]/PlGF ratio).

**RESULTS:**

A total of 1252 pregnant individuals were included. Abnormal initial PlGF-based test identified a more severe phenotype of preeclampsia, at increased risk of adverse maternal and perinatal outcomes. Repeat testing was not significantly associated with clinical benefit in women with abnormal initial results. Of women with a normal initial result, 20% developed preeclampsia, with the majority at least 3 to 4 weeks after initial presentation. Repeat test results were more likely to change from normal to abnormal in symptomatic women (112/415; 27%) compared with asymptomatic women (163/890; 18%). A higher proportion of symptomatic women who changed from normal to abnormal were diagnosed with preeclampsia, compared with asymptomatic women.

**CONCLUSIONS:**

Our results do not demonstrate evidence of the clinical benefit of repeating PlGF-based testing if the initial result is abnormal. Judicious use of repeat PlGF-based testing to stratify risk may be considered at least 2 weeks after a normal initial test result, particularly in women who have symptoms or signs of preeclampsia.

**REGISTRATION:**

URL: https://www.isrctn.com/ISRCTN85912420; Unique identifier: ISRCTN85912420.

**NOVELTY AND RELEVANCE**

**What Is New?**

To our knowledge, the PARROT-2 trial (Repeat Placental Growth Factor-Based Testing in Women With Suspected Preterm Preeclampsia) was the first trial of repeat PlGF (placental growth factor)-based testing for suspected preeclampsia. This planned secondary analysis presents novel data on subgroups, exploring whether stratification according to initial PlGF-based test results and PlGF-based test type informs a repeat testing strategy.

**What Is Relevant?**

PlGF-based testing has transformed the diagnosis and risk stratification of women presenting with suspected preeclampsia. It is a common question from clinicians whether PlGF-based testing should be repeated, in women who have received a one-off PlGF-based test as part of assessment for suspected preeclampsia. UK National Guidance recommended further research exploring different scenarios in which repeat testing may be indicated.

**Clinical/Pathophysiological Implications?**

Repeat testing is not associated with clinical benefit in women with an abnormal or very abnormal initial PlGF-based test result. Repeat testing may be considered in women with a normal initial PlGF-based test result, on an indicated basis if a high level of clinical suspicion remains. There is no evidence to support routine, universal repeat testing in any of the subgroups.

Preeclampsia has been reported to affect 2.8% of the pregnant population.^1^ Suspected preeclampsia is far more common and accounts for a substantial proportion of the workload within maternity services.^2^ It can be challenging to diagnose and manage, given the variable clinical presentation and potential for unpredictable, rapid deterioration. Historic inability to predict adverse outcomes has led to unnecessarily high levels of intervention, including admission and iatrogenic preterm delivery.^3^ Better methods of risk stratification and targeted surveillance may reduce maternal and perinatal morbidity and mortality.

Distinct angiogenic biomarker profiles, with a low concentration of PlGF (placental growth factor) or a high ratio of antiangiogenic sFlt-1 (soluble fms-like tyrosine kinase) to PlGF, accurately predict preeclampsia necessitating expedited delivery in women with suspected disease.^4–6^ Trials have demonstrated that a one-off test when preeclampsia is first suspected improves clinical precision, reduces time to diagnosis, and reduces severe maternal adverse outcomes.^7,8^ The PARROT-2 trial (Placental Growth Factor Repeat Sampling for Reduction of Adverse Perinatal Outcomes in Women with Suspected Pre-eclampsia) of revealed versus concealed repeat PlGF-based testing demonstrated a significant reduction in time to diagnosis (19.1 [SD, 20.4] versus 22.5 [SD, 22.9]; mean difference, −3.79 [−7.10 to −0.47] days; *P*=0.025) but no significant association with a reduction in perinatal or maternal adverse outcomes.^9^

This planned secondary analysis aimed to answer whether there is a group of women who may benefit from repeat PlGF-based testing, if participants are stratified either by initial PlGF-based test result or by test type, QuidelOrtho PlGF testing or Roche sFlt-1/PlGF ratio testing.

## METHODS

### Data Sharing

The data set will be available to appropriate academic parties on request from the chief investigator (L.C.C.) in accordance with the data sharing policies of King’s College London, with input from the coinvestigator group where applicable.

This was a planned secondary analysis of the PARROT-2 trial.^10^ The PARROT-2 trial was an individual-level randomized controlled trial of repeat revealed PlGF-based testing, compared with usual care with repeat concealed PlGF-based testing, in women with suspected preterm preeclampsia (ISRCTN85912420), approved by the Cambridge East Research Ethics Committee (No. 19/EE/0322). Women and birthing people were recruited from 22 maternity units across England, Scotland, and Wales, with a singleton live fetus, between 22 weeks’ gestation and 35 weeks and 6 days’ gestation at the time of the initial PlGF-based test. All participants received an initial revealed PlGF-based test, in accordance with UK national guidance.^11^ Suspected preeclampsia was defined as at least 1 of new onset or worsening of existing hypertension, proteinuria, neurological symptoms, severe headache, epigastric or right upper quadrant, suspected fetal growth restriction, or abnormal maternal blood tests consistent with preeclampsia (thrombocytopenia, hemolysis, hepatic, or renal dysfunction). Women with a clinician-confirmed, documented diagnosis of preeclampsia were not eligible. Participants provided written consent. Women were individually randomized to repeat revealed PlGF-based testing, or usual care with repeat concealed testing, with minimization according to the maternity unit, the primary indication for testing (hypertension or other) and gestational age at randomization (22^+0^–27^+6^, 28–31^+6^, >32^+0^).

Maternity units implementing either the QuidelOrtho PlGF test or the Roche sFlt-1/PlGF ratio testing were eligible to participate in the trial. Women provided blood samples at the same time as routine clinical blood tests where possible, to a maximum of 4× during their pregnancy. Symptoms or signs of suspected preeclampsia were recorded at repeat testing visits, where possible. Repeat testing was implemented with a management algorithm (Figure S1). It was emphasized to participating sites that there are insufficient data regarding PlGF-based testing beyond 37 weeks of gestation and in confirmed preeclampsia and that care in these situations should follow National Guidelines.^11^ The repeat testing schedule was

- PlGF <100 pg/mL or sFlt-1/PlGF >38 (test abnormal): weekly sampling (±2 days).
- PlGF ≥100 pg/mL or sFlt-1/PlGF ≤38 (test normal), sampling every 2 weeks (±7 days) if asymptomatic or earlier if presenting again with symptoms or signs of preeclampsia at least 7 days from the last test.

Concealed samples were spun, stored at −80 °C, and processed after the last participant had delivered, to assess the test performance of repeat tests for predicting preeclampsia.

### Outcomes

Outcomes were collected until primary hospital discharge of the mother and infant (or the end of the trial for 2 infants who were not discharged by the end of the trial). The primary outcome was a perinatal composite outcome of stillbirth, early neonatal death, and neonatal unit admission. Secondary outcomes are available in full in the published protocol.^10^ Tested secondary perinatal outcomes included gestational age at delivery, preterm birth before 37 weeks of gestation, and before 34 weeks of gestation. Descriptive outcomes included birthweight centile (using Intergrowth-21st standards), birthweight <10th centile, and survival to discharge without severe morbidity^12^ (defined as survival to discharge without brain injury, bronchopulmonary dysplasia, severe necrotizing enterocolitis, retinopathy of prematurity, or late-onset sepsis). Tested secondary maternal outcomes included a severe maternal adverse outcome composite,^13^ severe hypertension >160 mm Hg, cesarean delivery (compared with vaginal delivery), proportion of participants diagnosed with preeclampsia,^14^ and time to diagnosis of preeclampsia from initial PlGF-based test. These outcomes match those used for the primary trial analysis.

### Sample Size

The sample size was determined to be 1208 participants for the main PARROT-2 trial. All participants fulfilling eligibility criteria, and with outcome data, were included in this secondary analysis.

### Statistical Analysis

For the secondary analysis stratified by initial PlGF-based test result, women were stratified into the following predetermined groups, as previously described^5,15^: PlGF ≥100 pg/mL or sFlt-1/PlGF ≤38, test normal; PlGF 12 to 99 pg/mL or sFlt-1/PlGF >38 to <85, test abnormal; PlGF <12 pg/mL or sFlt-1/PlGF ≥85, test very abnormal. We compared how outcomes were influenced by trial arm in each subgroup, to determine whether there is a group of women who benefit from repeat revealed PlGF-based testing. For the secondary analysis stratified by PlGF-based test type, women were stratified according to whether they received QuidelOrtho PlGF testing or Roche sFlt-1/PlGF ratio testing.

The analysis was by the intention-to-treat principle, with randomized participants analyzed in their original groups. Analyses were carried out using a 2-sided type 1 error rate of 0.05. The binary composite of stillbirth, early neonatal death or neonatal unit admission was analyzed using binomial regression with a log link. Results are presented as unadjusted risk ratios, with 95% CIs. Tested secondary outcomes were analyzed using log-binomial regression models with a log link and results were presented as unadjusted risk ratios with 95% CIs. Continuous outcomes were analyzed using linear regression with log transformations as necessary. A full statistical analysis plan has been published.^10^

We performed an additional exploratory analysis of women with a normal initial PlGF-based test result, to evaluate whether these data could inform a repeat testing strategy in these women. This included the presence of symptoms or signs of preeclampsia at repeat testing visits, changing the PlGF-based test category, and diagnosis of preeclampsia, in 2-week windows. No formal significance testing has been done on this exploratory analysis.

Analyses were done with Stata version 17 (StataCorp, College Station, TX).

## RESULTS

A total of 1252 women were included in this analysis: 625 in the repeat revealed PlGF-based testing group and 627 in the repeat concealed group (Figure S2). One woman in the concealed group was lost to follow-up. For the analysis stratified by initial test result, 716 participants (57.2%) had a normal initial PlGF-based test result, 335 participants (26.8%) had an abnormal initial test result, and 201 participants (16.1%) had a very abnormal initial test result. For the analysis stratified by test type, 789 participants (63.0%) received QuidelOrtho PlGF testing, and 463 participants (37.0%) received Roche sFlt-1/PlGF ratio testing.

### Clinical Characteristics Stratified by First Test Result

Baseline characteristics are shown in Table 1 (including 1 woman who was later lost to follow-up). A smaller proportion of women with a very abnormal initial result were prescribed prophylactic aspirin (82/201, 40.8%) compared with those with a normal initial result (441/716, 61.5%).

**Table 1.**
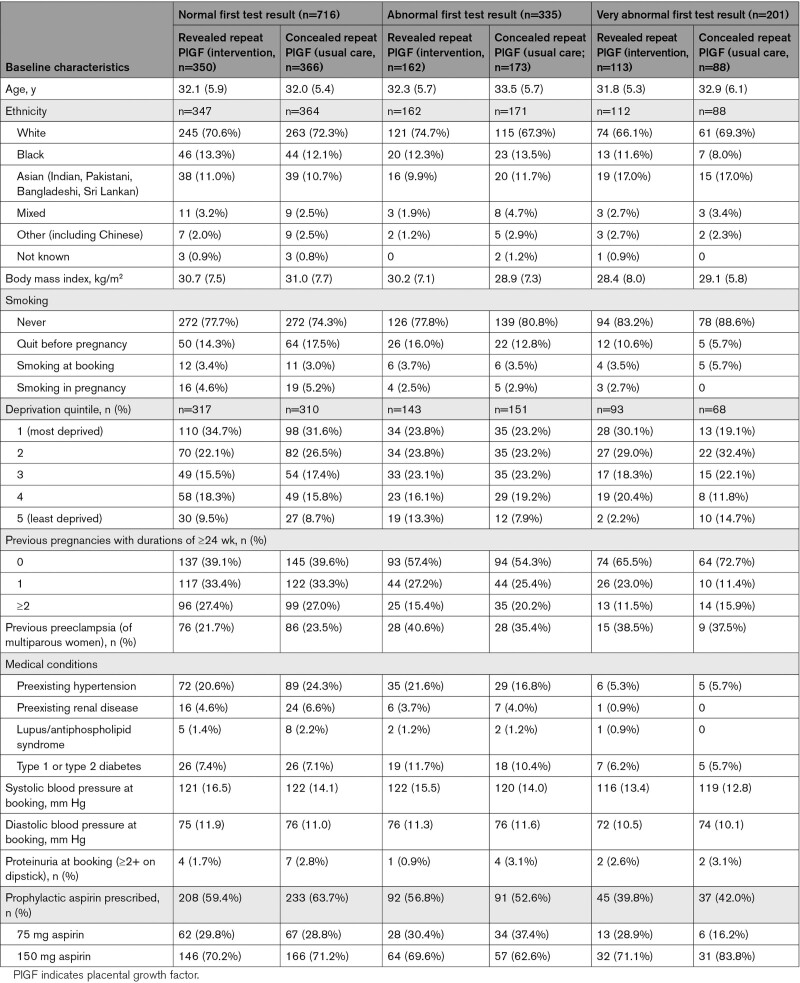
Baseline Demographics and Clinical Characteristics, Stratified by First Test Result

Women with a very abnormal initial PlGF-based test had worse signs of preeclampsia, with higher systolic and diastolic blood pressure, more significant proteinuria, and more fetal growth abnormalities on ultrasound (Table 2). In women randomized to revealed testing compared with concealed testing, 54.0% and 44.3% of women were admitted to the hospital with a very abnormal initial result, compared with 13.1% and 10.9% with a normal initial result.

**Table 2.**
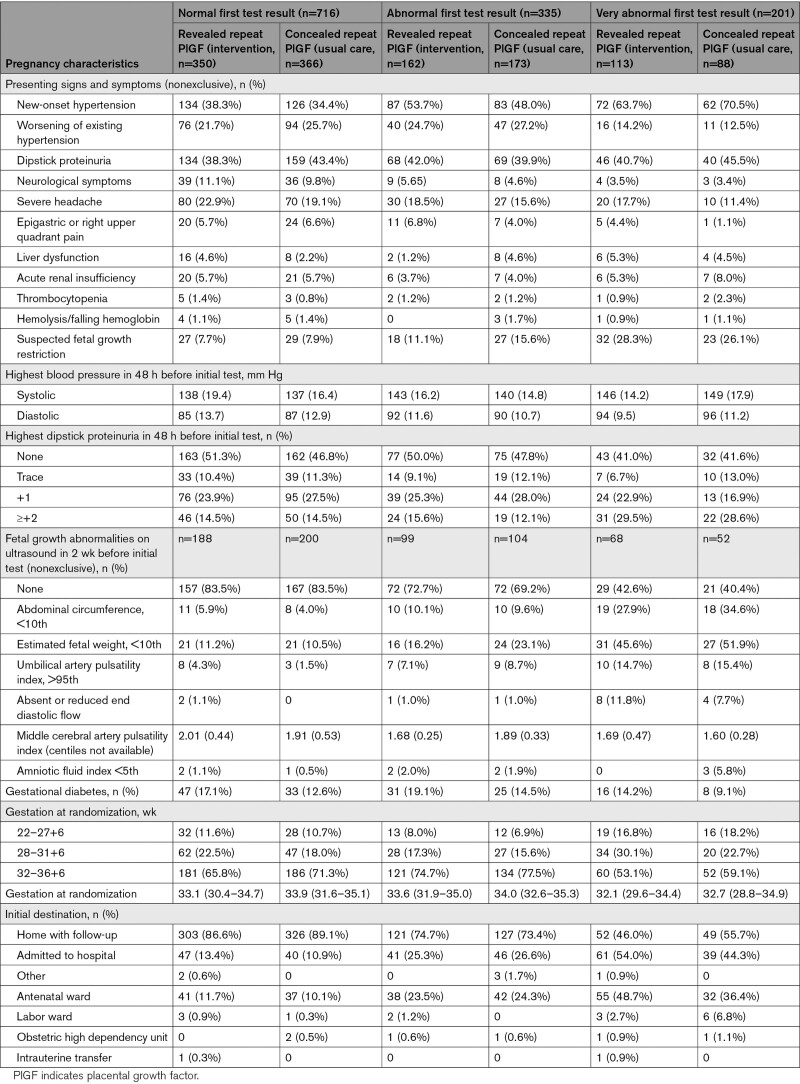
Pregnancy Characteristics at First PlGF-Based Test, Stratified by First Test Result

### Perinatal Outcomes

Very abnormal initial PlGF-based test results identified a more severe phenotype of preeclampsia, with worse perinatal outcomes (Table 3). In the revealed repeat testing group compared with the concealed testing group, the primary perinatal composite outcome was 69.0% in both groups with a very abnormal initial result; 37.0% versus 30.1% (relative risk [RR], 1.23 [0.91–1.67]; *P*=0.176) in women with an abnormal initial result; and 16.3% versus 16.9% (RR, 0.96 [0.69–1.34]; *P*=0.814) in women with a normal initial result. Five of the 7 perinatal deaths occurred in the group with a very abnormal initial result.

**Table 3.**
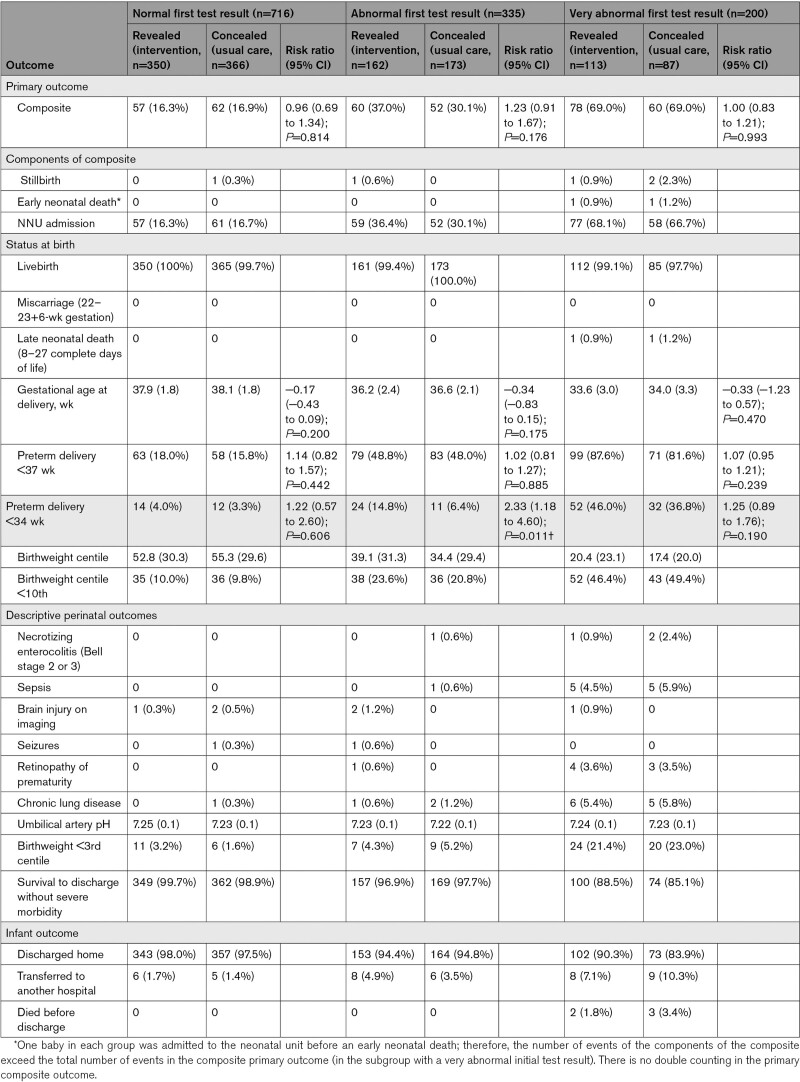
Primary Outcome and Secondary Perinatal Outcomes, Stratified by First Test Result

In the revealed group compared with the concealed group, the preterm birth rate before 34 weeks of gestation was significantly increased in women with an abnormal initial result (14.8% versus 6.4%; RR, 2.33 [95% CI, 1.18–4.60]; *P*=0.011), but not significantly increased in women with a very abnormal initial result (46.0% versus 36.8%; RR, 1.25 [95% CI, 0.89–1.76]; *P*=0.190) nor in women with a normal initial result (4.0% versus 3.3%; RR, 1.22 [95% CI, 0.57–2.60]; *P*=0.606). In the revealed group compared with the concealed group, morbidity-free survival to discharge was 88.5% versus 85.1% in the group with a very abnormal initial result, 96.9% versus 97.7% in the group with an abnormal result and 99.7% versus 98.9% in the group with a normal initial result (Table 3).

### Maternal Outcomes

Very abnormal initial PlGF-based test results similarly identified more severe maternal disease. Of women with a very abnormal initial result, 86% were diagnosed with preeclampsia and 5% had a severe maternal adverse outcome (Table 4). Longitudinal trajectories in PlGF and sFlt-1/PlGF results are demonstrated in the Figure and Figure S3, demonstrating a flat profile of sFlt-1/PlGF ratio in women with an initial abnormal sFlt-1/PlGF ratio who were diagnosed with preeclampsia. Among participants with a normal initial test result, in the revealed group Cesarean delivery was 61.1% compared with 53.8% in the concealed group (RR, 1.14 [95% CI, 1.0–1.29]; *P*=0.045). There were no other significant differences in maternal outcomes between subgroups.

**Table 4.**
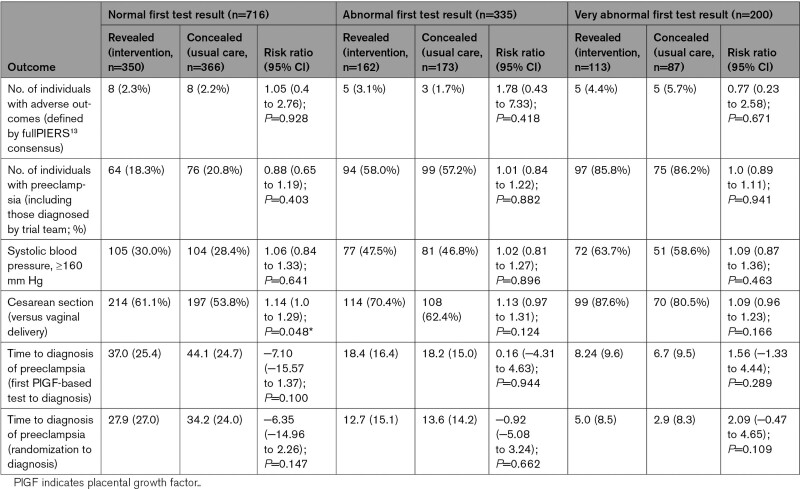
Secondary Maternal Outcomes With Comparisons, Stratified by First Test Result

**Figure.**
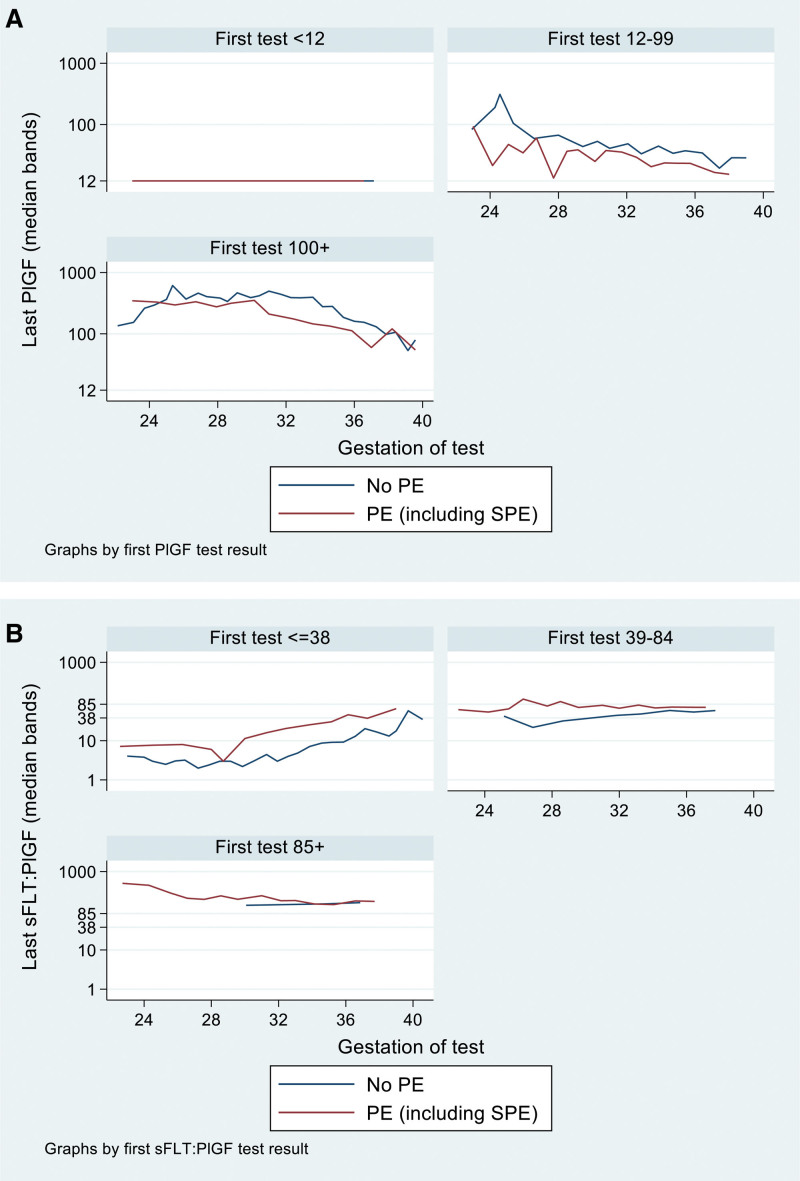
**Longitudinal measurements of PlGF (placental growthfactor) and sFlt-1 (soluble fms-like tyrosine kinase-1)/PlGF across gestation. A**, Median bands of longitudinal measurements of PlGF (pg/mL) across gestation, in women with at least 1 repeat test, stratified by initial test result, and final diagnosis of preeclampsia (PE; revealed and concealed groups). **B**, Median bands of longitudinal measurements of sFlt-1/PlGF across gestation, in women with at least 1 repeat test, stratified by initial test result, and final diagnosis of preeclampsia (revealed and concealed groups). SPE indicates superimposed pre-eclampisa.

Of women with a normal initial result, 14/716 women (2.0%) developed preeclampsia with delivery within 21 days and 32/716 (4.5%) within 28 days (Table 5). In comparison, 122/200 (61.0%) women with a very abnormal result (PlGF, <12 pg/mL or sFlt-1/PlGF, >85) developed preeclampsia within 21 days (Table 5).

**Table 5.**
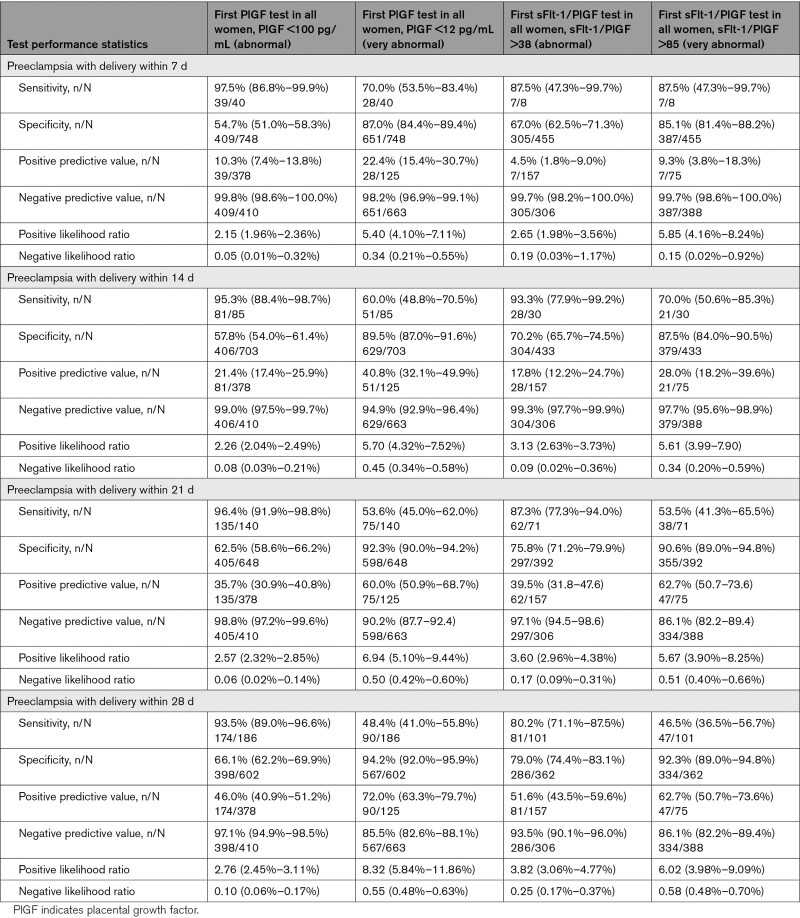
Test Performance of PlGF Test in Predicting Preeclampsia With Delivery Within 7, 14, 21, and 28 Days, in Women up to 35+6 Weeks of Gestation Presenting With Suspected Preeclampsia (for Women Receiving Repeat Concealed PlGF-Based Testing Only)

Of women with a normal initial result, 19.6% (140/716) received a final diagnosis of preeclampsia (including adjudicated diagnosis; Table 4). The Figure and Figure S3 demonstrate that women with an initial normal test who went on to develop preeclampsia had a decrease in PlGF or increase in sFlt-1/PlGF earlier than women who did not develop preeclampsia. In our exploratory analysis of repeat testing in 2-week windows (Table S1), 30% to 40% of women had symptoms or signs of preeclampsia at repeat testing visits, 16% to 32% of women changed from normal to abnormal PlGF-based test category, 2% to 6% changed to very abnormal PlGF-based test category, and 5% to 8% of women were diagnosed with preeclampsia in each 2-week window. Results were similar in the concealed group only (Table S2). A higher proportion of women with symptoms or signs of preeclampsia at repeat testing visits changed the PlGF-based test category (27.0% with symptoms or signs of preeclampsia versus 18.3% of asymptomatic women; Tables S3 and S4). There was a greater difference between the proportion of women with an abnormal test result with a diagnosis of preeclampsia compared with those with a normal test with a diagnosis of preeclampsia, in the group with symptoms and signs of preeclampsia, versus the asymptomatic group (Tables S5 and S6). For example, at 2 to 4 weeks, 57.6% of symptomatic women with an abnormal test were diagnosed with preeclampsia versus 19.3% of symptomatic women with a normal test, compared with 25.5% of asymptomatic women with an abnormal test versus 16.4% of asymptomatic women with a normal test (Table S5). Results were similar when restricting analysis to the concealed group only (Tables S4 and S6). We performed a subgroup analysis of primary and secondary end points for participants with a normal initial test, restricting inclusion to participants who received their first repeat test >2 weeks after the first test; this demonstrated similar results (Tables S7 and S8).

### Time to Diagnosis and Delivery

In the revealed group compared with the concealed group, time to diagnosis was reduced by 7 days in women with a normal initial result (mean, 37.0 [25.4] versus 44.1 [24.7] days; mean difference, −7.1 [−15.57 to 1.37] days; *P*=0.100; Table 4).

Time to delivery with preeclampsia was shorter in women with a normal initial test and abnormal repeat test (Table S9; median 20.0 [interquartile range, 18.0–24.0] days for PlGF testing [n=10], median 13.0 [interquartile range, 7.0–31.0] days for sFlt-1/PlGF testing [n=5]) compared with women in whom the repeat PlGF-based test remained normal (median, 34.0 [interquartile range, 20.0–53.0] days for PlGF testing [n=25]; median, 35.0 [interquartile range, 26.0–39.5] for sFlt-1/PlGF testing [n=20]). Time to delivery for any reason is shown in Table S10.

### Clinical Characteristics Stratified by PlGF-Based Test Type

Baseline characteristics and clinical characteristics at the time of the first PlGF-based test are shown in Tables S11 and S12. In the sFlt-1/PlGF testing group compared with the PlGF testing group, there was a higher proportion of participants in the most deprived quintile and a higher proportion of 150 mg aspirin compared with 75 mg (93% versus 54%).

### Perinatal Outcomes

The proportion of infants with the primary composite outcome was similar in the revealed PlGF testing group (30.9%), and the revealed (31.6%) and concealed (33.5%) sFlt-1/PlGF testing groups, with a surprising low event rate in the concealed PlGF testing group (24.5%; RR, 1.26 [95% CI, 1.00–1.58]; *P*=0.046; Table S13). In the revealed PlGF testing group compared with the concealed PlGF testing group, there was an increase in neonatal unit admission (30.6% versus 24.0%; RR, 1.28 [95% CI, 1.01–1.61]; *P*=0.037), reduction in gestational age at delivery (36.8 versus 37.2 days; mean difference, −0.45 [95% CI, −0.81 to 0.09] days; *P*=0.014) and increase in the rate of preterm birth before 34 weeks of gestation (13.5% versus 6.8%; RR, 1.98 [95% CI, 1.27–3.08]; *P*=0.002); similar results were not seen in the sFlt-1/PlGF group. However, the lowest preterm birth rate before 34 weeks of gestation was in the concealed PlGF testing group (6.8% in the concealed group versus 13.5% in the revealed group; RR, 1.98 [95% CI, 1.27–3.08], *P*=0.002). There was a chance imbalance in the proportion of participants with a very abnormal initial PlGF test, with 18.9% in the revealed testing group, compared with 13.1% in the concealed testing group; this may account for some of these differences. Morbidity-free survival to discharge was similar between groups.

### Maternal Outcomes

Repeat revealed testing was significantly associated with an increase in Cesarean delivery in the PlGF testing group (69.1% versus 58.6% in the revealed versus concealed groups; RR, 1.18 [95% CI, 1.06–1.31]; *P*=0.002), but not in the sFlt-1/PlGF testing group (Table S14).

### Test Performance

Test performance for prediction of preeclampsia with delivery within 14 days is demonstrated in Table 5, stratified by initial test result and test type. PlGF ≥100 pg/mL had a negative predictive value of 99.0% (95% CI, 97.5%–99.7%); sFlt-1/PlGF >38 had a negative predictive value of 99.3% (95% CI, 97.7%–99.9%). PlGF <12 pg/mL had a positive predictive value for predicting preeclampsia with delivery within 14 days of 40.8% (95% CI, 32.1%–49.9%), rising to 72.0% (95% CI, 63.3%–79.7%) for 28 days; sFlt-1/PlGF >85 had a positive predictive value for predicting preeclampsia with delivery within 14 days of 28.0% (95% CI, 18.2%–39.6%), increasing to 62.7% (95% CI, 50.7%–73.6%) for 28 days.

## DISCUSSION

This secondary analysis of a large, multicenter trial of repeat PlGF-based testing aimed to answer whether there are subgroups of women who may benefit from repeat PlGF-based testing. Our results do not demonstrate evidence of clinical benefit in repeating PlGF-based testing if the initial result is abnormal. There may be benefit in repeat testing if the initial result is normal, particularly after at least 2 weeks, and in women who have new symptoms and signs of preeclampsia. Twenty percent (140/716) were diagnosed with preeclampsia, and 34% of women changed the PlGF category by their final test. Test performance for predicting preeclampsia with delivery remained high for 3 to 4 weeks: 2.0% and 4.5% of women were diagnosed with preeclampsia within 3 or 4 weeks of the initial normal test, respectively. Exploratory analysis showed a higher proportion of women who changed the test category were diagnosed with preeclampsia, although not all women with a changing test result were diagnosed with preeclampsia, and a smaller proportion of women with a normal test received a diagnosis of preeclampsia. In women changing the PlGF-based test category, a higher proportion were diagnosed with preeclampsia if they had symptoms or signs of preeclampsia, suggesting symptoms or signs are indicative of changing category and evolving preeclampsia. In the subgroup analysis of participants with a normal initial test with repeat testing >2 weeks after the initial test, there was no significant clinical benefit, but adverse events were rare, and this exploratory post hoc analysis may be underpowered. This is an exploratory analysis with small numbers in each group; nevertheless, this could inform surveillance strategies and there may be a rationale for repeating PlGF-based testing after at least 2 weeks, in a subset of high-risk women with a normal initial result, particularly if there is continued clinical suspicion. In women with a normal initial result, median time to diagnosis was 7 days shorter in the revealed group compared with the concealed group. Although this did not reach significance, the absence of any difference in other groups implies that the overall significant reduction in time to diagnosis in the main trial analysis (−3.8 [95% CI, −7.1 to −0.5] days; *P*=0.025) is driven by this group.^9^

Abnormal angiogenic biomarker concentration at the time of first presentation with suspected preeclampsia accurately identifies a more severe phenotype of preeclampsia, with worse maternal and neonatal outcomes. This has been previously demonstrated but is worth describing in this large cohort, and to put into context the effect of the intervention of repeat PlGF-based testing. In women with an abnormal or very abnormal initial result, repeat testing was not significantly associated with reduced adverse outcomes or reduced time to diagnosis. It is unclear why a higher proportion of participants were admitted after an initial very abnormal test in the repeat revealed arm (54.0%), compared with the concealed arm (44.3%). It is possible that repeating the test influenced clinical decision-making regarding admission. However, this was not a prespecified analysis, and, therefore, it is difficult to interpret, and maybe a chance finding. Furthermore, there was no difference in total time on antenatal ward between groups.^9^

Repeat testing was significantly associated with increased preterm birth before 34 weeks of gestation in women with an initial abnormal test result (RR, 2.33 [95% CI, 1.18–4.60]; *P*=0.011; Table 3). The mechanism of this is unclear but may be related to clinician behavior in response to repeated abnormal results, as tests rarely normalized.^9^ Indications for preterm delivery have previously been presented,^9^ and the clinical management algorithm (Figure S1) emphasized that abnormal biomarker concentration alone should not be considered an indication for delivery. It is possible that in a different setting with a higher prevalence of stillbirth (such as low- and middle-income settings), iatrogenic preterm birth may prevent stillbirth and the components of the composite might go in opposite directions, with a reduction in perinatal death and an increase in neonatal unit admission, but this was not demonstrated in our study in a high-income setting with a low prevalence of perinatal death (7/1251 participants, 0.56%). The stillbirth rate of 0.4% observed in this study of high-risk women with suspected preeclampsia, all receiving an initial PlGF-based test according to national guidance,^11,16^ is the same as the background population stillbirth rate; this is reassuring and supports the importance of initial PlGF-based testing informing risk stratification and management.

To our knowledge, this is the largest randomized trial of repeat revealed PlGF-based testing compared with usual care with repeat concealed testing, in women with suspected preterm preeclampsia. Strengths of the study include broad inclusion criteria and diverse participants, both in terms of demography and disease severity, enhancing the generalizability of our findings to other high-income settings. The large study size enabled the evaluation of the trial results according to stratification by initial test result and test type. We have previously presented data investigating the effect of gestational age, and this demonstrated no evidence of the benefit of repeating the test being different according to gestation.^9^

Our study has some limitations. Stratification into 6 subgroups resulted in smaller numbers and lower statistical power, meaning that we may be underpowered to detect significant differences in outcomes. Due to small numbers, convergence was not achieved, and unadjusted risk ratios were supplied. As there was a low prevalence of adverse outcomes in our study, these results may not be generalizable to high-burden, low-income settings. Different maternity units have adopted either the QuidelOrtho PlGF test or Roche sFlt-1/PlGF ratio testing according to unique barriers and facilitators to implementation, and this site variation resulted in distinct populations limiting direct comparison between these groups. However, test performance for prediction of preeclampsia with delivery within 14 days was comparable between the 2 tests. According to the protocol, PlGF-based testing was performed after 37 weeks of gestation, and after a diagnosis of preeclampsia. Existing guidance does not recommend testing in these situations. This was emphasized to sites, with a recommendation that care should continue to follow National Guidance on Hypertension in Pregnancy. In total, 378 women received repeat tests after 37 weeks of gestation (of a total of 2583 repeat testing visits, 14.6%). The protocol stipulated repeat testing in women asymptomatic for suspected preeclampsia. Although testing might not be repeated in all asymptomatic women in clinical practice, it is evident that clinicians are using repeat testing in multiple clinical settings, including asymptomatic patients, due to ongoing clinical uncertainty. This trial was a pragmatic, real-world randomized controlled trial, designed to address this uncertainty and, therefore, the protocol recommended repeat testing of asymptomatic women. Data on the gestation of commencing aspirin and adherence were not available, but this was not the focus of this study.

Risk stratification by initial PlGF-based test is consistent with previous studies of angiogenic biomarkers. In 1006 women included in the stratified analysis of the PARROT-1 trial^15^ of revealed versus concealed PlGF testing, PlGF <100 pg/mL identified women with more marked hypertension, increased adverse outcomes, and preterm birth. In 1112 women included in the secondary analysis of PETRA (Preeclampsia Triage by Rapid Assay Trial)^17^ of concealed PlGF testing, low PlGF <100 pg/mL was significantly associated with composite maternal adverse outcomes (6.2% versus 1.9%; adjusted RR, 3.6 [95% CI, 1.7–8.0]) and composite neonatal adverse outcomes (9.2% versus 0.8%; adjusted RR, 17.2 [95% CI, 5.2–56.3]). Our trial confirms that adverse outcomes are infrequent in women with normal initial PlGF-based test results and more common in women with abnormal and very abnormal initial results. However, our study contrasts with previous studies demonstrating that the delta between tests is associated with faster deterioration.^18,19^ To our knowledge, ours is the largest study of repeat PlGF-based testing, and we have demonstrated flat longitudinal biomarker profiles in women with an initial abnormal sFlt-1/PlGF ratio who were diagnosed with preeclampsia or who developed severe adverse outcomes. Previously published data have included small numbers of participants with confirmed preeclampsia, and the delta was small (delta, 48.97 at repeat sFlt-1/PlGF testing at 3 weeks; n=10 women with preeclampsia).

The analysis stratified by test type demonstrated some surprising results. The primary outcome, driven by neonatal unit admission, was similar in the PlGF revealed group, the sFlt-1/PlGF revealed group, and the sFlt-1/PlGF concealed group, with a significantly lower prevalence in the PlGF concealed group (24.5% versus 30.9%; RR, 2.33 [95% CI, 1.00–1.58]; *P*=0.046). This is challenging to explain but may relate to the chance imbalance in very abnormal initial PlGF tests between groups or variation in clinical practice between hospitals implementing the QuidelOrtho PlGF and Roche tests. The populations were distinct as participants were recruited from different maternity units, and, therefore, this is not a direct comparison of PlGF versus sFlt-1/PlGF testing. McCarthy et al^20^ demonstrated that the area under the receiver operating curve is comparable for all currently recommended PlGF-based tests and small variations in sensitivity and specificity are likely related to distinct thresholds. To date, there has not been a direct comparison of the assays in a prospective study.

### Perspectives

To our knowledge, the PARROT-2 trial was the first randomized trial of repeat PlGF-based testing for suspected preterm preeclampsia. This planned secondary analysis has stratified participants by initial PlGF-based test category and by PlGF-based test type. This demonstrates that there is no clinical benefit and significantly increased preterm birth before 34 weeks of gestation, associated with repeat testing in women with an initial abnormal PlGF-based test. Contrary to published smaller studies, we have demonstrated flat longitudinal sFlt-1/PlGF profiles in women with abnormal or very abnormal initial results. There was no significant harm or benefit associated with repeat testing in women with an initial normal result; 30% to 40% of these women changed the PlGF-based test category on repeat tests and 20% developed preeclampsia, with a higher proportion in symptomatic women.

At present, there are insufficient data to recommend variable thresholds depending on ethnicity or other maternal factors, and this was a trial of repeat PlGF-based testing according to UK guidance with recommended thresholds. However, we are planning to investigate differences in PlGF thresholds according to ethnicity in this large cohort. Future research should also include a cost-effectiveness analysis of repeat PlGF-based testing. Evaluation of PlGF-based testing and repeat PlGF-based testing in high-burden, low-income settings is necessary; risk stratification and timely action in women at high risk of adverse outcomes, including appropriate iatrogenic preterm birth, may improve global maternal and perinatal outcomes.

### Conclusions

The results of this stratified analysis of the PARROT-2 trial emphasize that PlGF-based testing accurately identifies a more severe phenotype of preeclampsia. Exploratory analysis suggests there may be a place for judicious repeat testing in women with an initial normal test result, after at least 2 weeks from the initial test and in women who present again with new symptoms and signs of preeclampsia.

## ARTICLE INFORMATION

### Acknowledgments

The authors thank the independent Trial Steering Committee (Dr Lucy Mackillop [chair], Baskaran Thilaganathan, Dr Kylie Watson, and Sarah Findlay) and the independent Data Monitoring Committee (Dr Katherine Tucker [chair], Baskaran Thilaganathan, and Dr Ushma Galal). The authors thank all women and birthing people who participated in the PARROT-2 trial (Repeat Placental Growth Factor-Based Testing in Women With Suspected Preterm Preeclampsia; women and birthing people who participated in the PARROT-2 trial as initially stated; they are subsequently referred to as women or individuals). PARROT-2 Trial Group Collaborators: Carolyn Gill, Sian McDonnell, Beth Peers, Angela Yulia, Orla Ferry, Martin Maher, Francis Pickering, Annabel Smith, Hilary Thompson, Sambita Basak, Lucy Dudgeon, Jo Ficquet, Mel Rich, Clare O’Brien, Seren Willson, Nikolaos Chados, Linda Bishop, Rachna Bahl, Brittany Smart, Rita Arya, Lindsay Roughley, Anku Mehta, Deniesha Campbell, Jo Girling, Grace Ryan, Lauren Trepte, Chandrima Biswas, Chinwe Obiozo, Lynda Verghese, Ashwin Ahuja, Sarah Davies, Katie Morris, Jessica Davison, Maeve Regan, Jenny Myers, Natalie Barry, Mel McBean, Jacqui Jennings, Andrew Sharp, Siobhan Holt, Laura Stirrat, Elaine Jack, Mihraban Bapir, Sharon Gowans, Hazel Alexander, Kim Hinshaw, and Lesley Hewitt.

### Author Contributions

A.H. Shennan and L.C. Chappell conceived the study. L. Webster, K.E. Duhig, J. Myers, C. Battersby, P.T. Seed, K. Clark, M. Green, A.H. Shennan, and L.C. Chappell were involved in securing funding for the study. A. Hurrell, J. Sparkes, L. Webster, Z. Vowles, A. Brockbank, and L.C. Chappell coordinated the study conduct and data collection. A. Hurrell and P.T. Seed did the study analyses, supervised by L.C. Chappell. R.M. Hunter did the health economic analysis. A. Hurrell, L. Webster, and L.C. Chappell wrote the article, with assistance from J. Sparkes, K.E. Duhig, P.T. Seed, J. Myers, C. Battersby, and A.H. Shennan. All authors approved the final version of the manuscript.

### Sources of Funding

The trial was funded by the Jon Moulton Charitable Foundation and Tommy’s. Funding for (PlGF) placental growth factor-based tests was also received from the Guy’s and St Thomas’ NIHR Biomedical Research Centre and Roche.

### Disclosures

A.H. Shennan has received funds from Perkin Elmer (Revvity) and QuidelOrtho for expenses to meetings and has received money from Roche as a consultant on strategy. The other authors report no conflicts.

## Supplementary Material


