# Supplementary material for: Repeat Placental Growth Factor-Based Testing in Women With Suspected Preterm Preeclampsia: A Stratified Analysis of the PARROT-2 Trial
Source: Hypertension. 2024 May 6;81(7):1561–73. doi: 10.1161/HYPERTENSIONAHA.123.22411 (PMC11177603; doi:10.1161/HYPERTENSIONAHA.123.22411)

Repeat placental growth factor-based testing in women with suspected preterm pre-eclampsia: a stratified analysis of the PARROT-2 trial

Alice Hurrell, Louise Webster, Jenie Sparkes, Cheryl Battersby, Anna Brockbank, Katherine Clark, Kate E Duhig, Carolyn Gill, Marcus Green, Rachael M Hunter, Paul T Seed, Zoe Vowles, Jenny Myers, Andrew H Shennan, Lucy C Chappell, on behalf of the PARROT-2 trial group [†]

Short title: Stratified analysis of the PARROT-2 trial

Department of Women and Children's Health, School of Life Course Sciences, King's College London, London UK

(A Hurrell MRCOG, L Webster PhD, J Sparkes BSc, A Brockbank MSc, C Gill PhD, PT Seed CStat, K Clark MRes, Z Vowles MSc, Prof A H Shennan MD, Prof L C Chappell PhD);

Maternal & Fetal Health Research Centre, Division of Developmental Biology and Medicine, School of Medical Sciences, Faculty of Biology, Medicine and Health, University of Manchester, Manchester Academic Health Science Centre, Manchester M13 9PL, United Kingdom

(K E Duhig PhD, Prof J Myers PhD);

Neonatal Medicine, School of Public Health, Faculty of Medicine, Imperial College London Chelsea and Westminster Hospital campus, 369 Fulham Road, London SW10 9NH

(C Battersby PhD);

Action on Pre-eclampsia, Evesham, UK

(M Green);

Institute of Epidemiology and Health Care, University College London, UK

(Prof R M Hunter MSc)

Correspondence to*:* Prof Lucy Chappell, Department of Women and Children's Health, School of Life Course Sciences, King's College London, Westminster Bridge Road, London, SE1 7EH, UK

Email: lucy.chappell@kcl.ac.uk

Contents

Table S1. Proportion of participants with a normal initial PlGF-based test result, with: a) new symptoms and signs of pre-eclampsia, b) changing to abnormal or very abnormal test result, c) diagnosis of pre-eclampsia, in 2-week windows (concealed and revealed groups combined, QuidelOrtho PlGF and Roche sFlt-1/PlGF tests) **4**

Table S2. Proportion of participants with a normal initial PlGF-based test result, with: a) new symptoms and signs of pre-eclampsia, b) changing to abnormal or very abnormal test result, c) diagnosis of pre-eclampsia, in 2-week windows (concealed group and revealed group separately, QuidelOrtho PlGF and Roche sFlt-1/PlGF tests) **6**

Table S3. Proportion of participants with a normal initial PlGF-based test result who have changed PlGF-based test category, by presence of new symptoms / signs, in 2-week windows of women **9**

(concealed and revealed groups combined, QuidelOrtho PlGF and Roche sFlt-1/PlGF testing) **9**

Table S4. Proportion of participants with a normal initial PlGF-based test result who have changed PlGF-based test category, by presence of new symptoms / signs, in 2-week windows of women **10**

(concealed group only, QuidelOrtho PlGF and Roche sFlt-1/PlGF testing) **10**

Table S5. Relationship between abnormal repeat PlGF-based test result and diagnosis of PE, in 2-week windows in participants with a normal initial PlGF-based test result, with and without new symptoms and signs of pre-eclampsia at repeat testing visit (concealed and revealed groups combined, QuidelOrtho PlGF and Roche sFlt-1/PlGF) **11**

Table S6. Relationship between abnormal repeat PlGF-based test result and diagnosis of PE, in 2-week windows in participants with a normal initial PlGF-based test result, with and without new symptoms and signs of pre-eclampsia at repeat testing visit (concealed group only, QuidelOrtho PlGF and Roche sFlt-1/PlGF) **13**

Table S7: Primary outcome and secondary perinatal outcomes, for women with a normal first test result and repeat testing at least two weeks from the initial test **15**

Table S8: Secondary maternal outcomes with comparisons, stratified by first test result, for women with a normal first test result and repeat testing at least two weeks from the initial test **17**

Table S9: Time to delivery with preeclampsia (median, interquartile range) stratified by first PlGF concentration (for women receiving repeat concealed PlGF-based testing only) **18**

Table S10: Time to delivery for any reason (median, interquartile range) stratified by first PlGF concentration (for women receiving at least one repeat test, repeat concealed PlGF-based testing only) **19**

Table S11: Baseline Demographics and clinical characteristics, stratified by test type **20**

Table S12: Pregnancy characteristics at first PlGF-based test, stratified by test type 23
Table S13: Primary outcome and secondary perinatal outcomes, stratified by test type ...26
Table S14: Secondary maternal outcomes with comparisons, stratified by test type.........30

Table S1. Proportion of participants with a normal initial PlGF-based test result, with: a) **new symptoms and signs of pre-eclampsia, b) changing to abnormal or very abnormal test result, c) diagnosis of pre-eclampsia,** in 2-week windows (concealed and revealed groups **combined**, **QuidelOrtho PlGF and Roche sFlt-1/PlGF tests**)

a) Symptomatic for pre-eclampsia	Asymptomatic	Symptoms / signs of pre-eclampsia	
Repeat test < 2 weeks n=279	171 (61.3%)	108 (38.7%)	
Repeat test 2-4 weeks n=400	279 (69.8%)	121 (30.3%)	
Repeat test 4-6 weeks n=290	206 (71.0%)	84 (29.0%)	
Repeat test 6-8 weeks n=200	135 (67.5%)	65 (32.5%)	
Repeat test >8 weeks n=136	99 (72.8%)	37 (27.2%)	
**b) Change to abnormal test result**	**Normal**	**Abnormal test result**	**Very abnormal test results**
Repeat test < 2 weeks n=279	235 (84.2%)	38 (13.6%)	6 (2.2%)
Repeat test 2-4 weeks n=400	320 (80.0%)	70 (17.5%)	10 (2.5%)
Repeat test 4-6 weeks n=290	202 (69.7%)	73 (25.2%)	15 (5.2%)
Repeat test 6-8 weeks n=200	135 (67.5%)	53 (26.5%)	12 (6.0%)
Repeat test >8 weeks n=136	92 (67.6%)	41 (30.1%)	3 (2.2%)
**c) Diagnosis of pre-eclampsia**	**No diagnosis of pre-eclampsia**	**Diagnosis of pre-eclampsia**	
Repeat test < 2 weeks n=279	266 (95.3%)	13 (4.7%)	
Repeat test 2-4 weeks	378 (94.5%)	22 (5.5%)	

n=400		
Repeat test 4-6 weeks<br>n=290	269 (92.8%)	21 (7.2%)
Repeat test 6-8 weeks<br>n=200	184 (92.0%)	16 (8.0%)
Repeat test >8 weeks<br>n=136	129 (94.9%)	7 (5.1%)

Table S2. Proportion of participants with a normal initial PlGF-based test result, with: a) **new symptoms and signs of pre-eclampsia, b) changing to abnormal or very abnormal test result, c) diagnosis of pre-eclampsia,** in 2-week windows (concealed group and revealed group **separately**, **QuidelOrtho PlGF and Roche sFlt-1/PlGF tests**)

**Concealed only**			
**a) Symptomatic for pre-eclampsia**	**Asymptomatic**	**Symptoms / signs of pre-eclampsia**	
Repeat test < 2 weeks n=131	81 (61.8%)	50 (38.2%)	
Repeat test 2-4 weeks n=205	138 (67.3%)	67 (32.7%)	
Repeat test 4-6 weeks n=147	109 (74.2%)	38 (25.9%)	
Repeat test 6-8 weeks N=105	68 (64.8%)	37 (35.2%)	
Repeat test >8 weeks n=77	57 (74.0%)	20 (26.0%)	
**b) Change to abnormal test result**	**Normal**	**Abnormal test result**	**Very abnormal test results**
Repeat test < 2 weeks n=131	110 (84.0%)	18 (13.7%)	3 (2.3%)
Repeat test 2-4 weeks n=205	168 (82.0%)	32 (15.6%)	5 (2.4%)
Repeat test 4-6 weeks n=147	103 (70.1%)	40 (27.2%)	4 (2.7%)
Repeat test 6-8 weeks N=105	75 (71.4%)	25 (23.8%)	5 (4.8%)
Repeat test >8 weeks n=77	55 (71.4%)	20 (26.0%)	2 (2.6%)
**c) Diagnosis of pre-eclampsia**	**No diagnosis of pre-eclampsia**	**Diagnosis of pre-eclampsia**	
Repeat test < 2 weeks	124 (94.7%)	7 (5.3%)	

n=131			
Repeat test 2-4 weeks<br>n=205	193 (94.2%)	12 (5.9%)	
Repeat test 4-6 weeks<br>n=147	136 (92.5%)	11 (7.5%)	
Repeat test 6-8 weeks<br>N=105	97 (92.4%)	8 (7.6%)	
Repeat test >8 weeks<br>n=77	72 (93.5%)	5 (6.5%)	
**Revealed only**			
**Symptomatic for pre-eclampsia**	**Asymptomatic**	**Symptoms / signs of pre-eclampsia**	
Repeat test < 2 weeks<br>n=148	90 (60.8%)	58 (39.2%)	
Repeat test 2-4 weeks<br>n=195	141 (72.3%)	54 (27.7%)	
Repeat test 4-6 weeks<br>n=143	97 (67.8%)	46 (32.2%)	
Repeat test 6-8 weeks<br>N=95	67 (70.5%)	28 (29.5%)	
Repeat test >8 weeks<br>n=59	42 (71.2%)	17 (28.8%)	
**Change to abnormal test result**	**Normal**	**Abnormal test result**	**Very abnormal test results**
Repeat test < 2 weeks<br>n=148	125 (84.5%)	20 (13.5%)	3 (2.0%)
Repeat test 2-4 weeks<br>n=195	152 (77.9%)	38 (19.5%)	5 (2.6%)
Repeat test 4-6 weeks<br>n=143	99 (69.2%)	33 (23.1%)	11 (7.7%)
Repeat test 6-8 weeks<br>N=95	60 (63.2%)	28 (29.5%)	7 (7.4%)
Repeat test >8 weeks	37 (62.7%)	21 (35.6%)	1 (1.7%)

n=59			

**Diagnosis of pre-eclampsia**	**No diagnosis of pre-eclampsia**	**Diagnosis of pre-eclampsia**
Repeat test < 2 weeks<br>n=148	142 (96.0%)	6 (4.0%)
Repeat test 2-4 weeks<br>n=195	185 (94.9%)	10 (5.1%)
Repeat test 4-6 weeks<br>n=143	133 (93.0%)	10 (7.0%)
Repeat test 6-8 weeks<br>N=95	87 (91.6%)	8 (8.4%)
Repeat test >8 weeks<br>n=59	57 (96.6%)	2 (3.4%)

Table S3. Proportion of participants with a normal initial PlGF-based test result who have changed PlGF-based test category, by presence of new symptoms / signs, in 2-week windows of women (concealed and revealed groups **combined**, **QuidelOrtho PlGF and Roche sFlt-1/PlGF testing**)

**Asymptomatic for pre-eclampsia**	**Normal**	**Abnormal**	**Very abnormal**
Repeat test < 2 weeks n=171	147 (86.0%)	18 (10.5%)	6 (3.5%)
Repeat test 2-4 weeks n=279	232 (83.2%)	42 (15.1%)	5 (1.8%)
Repeat test 4-6 weeks n=206	150 (72.8%)	47 (22.8%)	9 (4.4%)
Repeat test 6-8 weeks n=135	100 (74.1%)	29 (21.5%)	6 (4.4%)
Repeat test >8 weeks n=99	70 (70.7%)	27 (27.3%)	2 (2.0%)
Total n=890	699 (78.5%)	163 (18.3%)	28 (3.1%)
**Symptomatic for pre-eclampsia**	**Normal**	**Abnormal**	**Very abnormal**
Repeat test < 2 weeks n=108	88 (81.5%)	20 (18.5%)	0
Repeat test 2-4 weeks n=121	88 (72.7%)	28 (23.1%)	5 (4.1%)
Repeat test 4-6 weeks n=84	52 (61.9%)	26 (31.0%)	6 (7.1%)
Repeat test 6-8 weeks n=65	35 (53.8%)	24 (36.9%)	6 (9.2%)
Repeat test >8 weeks n=37	22 (59.5%)	14 (37.8%)	1 (2.7%)
Total n=415	285 (68.7%)	112 (27.0%)	18 (4.3%)

Table S4. Proportion of participants with a normal initial PlGF-based test result who have changed PlGF-based test category, by presence of new symptoms / signs, in 2-week windows of women (concealed group only, QuidelOrtho PlGF and Roche sFlt-1/PlGF testing)

**Asymptomatic for pre-eclampsia**	**Normal**	**Abnormal**	**Very abnormal**
Repeat test < 2 weeks<br>n=81	70 (86.4%)	8 (9.9%)	3 (3.7%)
Repeat test 2-4 weeks<br>n=138	115 (83.3%)	20 (14.5%)	3 (2.2%)
Repeat test 4-6 weeks<br>n=109	80 (73.4%)	26 (23.9%)	3 (2.8%)
Repeat test 6-8 weeks<br>n=68	54 (79.4%)	12 (17.6%)	2 (2.9%)
Repeat test >8 weeks<br>n=57	43 (75.4%)	13 (22.8%)	1 (1.8%)
Total n=453	362 (79.9%)	79 (17.4%)	12 (2.6%)
**Symptomatic for pre-eclampsia**	**Normal**	**Abnormal**	**Very abnormal**
Repeat test < 2 weeks<br>n=50	40 (80.0%)	10 (20.0%)	0
Repeat test 2-4 weeks<br>n=67	53 (79.1%)	12 (17.9%)	2 (3.0%)
Repeat test 4-6 weeks<br>n=38	23 (60.5%)	14 (36.8%)	1 (2.6%)
Repeat test 6-8 weeks<br>n=37	21 (56.8%)	13 (35.1%)	3 (8.1%)
Repeat test >8 weeks<br>n=20	12 (60.0%)	7 (35.0%)	1 (5.0%)
Total n=212	149 (70.3%)	56 (26.4%)	7 (3.3%)

Table S5. Relationship between abnormal repeat PlGF-based test result and diagnosis of PE, in 2-week windows in participants with a normal initial PlGF-based test result, **with and without new symptoms** and signs of pre-eclampsia at repeat testing visit (concealed and revealed groups **combined**, **QuidelOrtho PlGF and Roche sFlt-1/PlGF**)

**Asymptomatic for pre-eclampsia**		
**Repeat test < 2 weeks**	**Normal test result**	**Abnormal test result**
No PE diagnosis	127 (86.4%)	15 (62.5%)
Diagnosis of PE	30 (13.6%)	9 (37.5%)
**Repeat test 2-4 weeks**	**Normal test result**	**Abnormal test result**
No PE diagnosis	194 (83.6%)	35 (74.5%)
Diagnosis of PE	39 (16.4%)	12 (25.5%)
**Repeat test 4-6 weeks**	**Normal test result**	**Abnormal test result**
No PE diagnosis	131 (87.3%)	38 (67.9%)
Diagnosis of PE	19 (12.7%)	18 (32.1%)
**Repeat test 6-8 weeks**	**Normal test result**	**Abnormal test result**
No PE diagnosis	88 (88.0%)	25 (71.4%)
Diagnosis of PE	12 (12.0%)	10 (28.6%)
**Repeat test > 8 weeks**	**Normal test result**	**Abnormal test result**
No PE diagnosis	63 (90.0%)	24 (82.8%)
Diagnosis of PE	7 (10.0%)	5 (17.2%)
**Symptomatic for pre-eclampsia**		
**Repeat test < 2 weeks**	**Normal test result**	**Abnormal test result**
No PE diagnosis	67 (76.1%)	11 (55.0%)
Diagnosis of PE	21 (23.9%)	9 (45.0%)

**Repeat test 2-4 weeks**	**Normal test result**	**Abnormal test result**
No PE diagnosis	71 (80.7%)	14 (42.4%)
Diagnosis of PE	17 (19.3%)	19 (57.6%)
**Repeat test 4-6 weeks**	**Normal test result**	**Abnormal test result**
No PE diagnosis	42 (80.8%)	15 (46.9%)
Diagnosis of PE	10 (19.2%)	17 (53.1%)
**Repeat test 6-8 weeks**	**Normal test result**	**Abnormal test result**
No PE diagnosis	28 (80.0%)	13 (43.3%)
Diagnosis of PE	7 (20.0%)	17 (56.7%)
**Repeat test > 8 weeks**	**Normal test result**	**Abnormal test result**
No PE diagnosis	19 (86.4%)	7 (46.7%)
Diagnosis of PE	3 (13.6%)	8 (53.3%)

Table S6. Relationship between abnormal repeat PlGF-based test result and diagnosis of PE, in 2-week windows in participants with a normal initial PlGF-based test result, **with and without new symptoms** and signs of pre-eclampsia at repeat testing visit (**concealed group only, QuidelOrtho PlGF and Roche sFlt-1/PlGF**)

**Asymptomatic for pre-eclampsia**		
**Repeat test < 2 weeks**	**Normal test result**	**Abnormal test result**
No PE diagnosis	58 (82.9%)	8 (72.7%)
Diagnosis of PE	12 (17.1%)	3 (27.3%)
**Repeat test 2-4 weeks**	**Normal test result**	**Abnormal test result**
No PE diagnosis	89 (77.4%)	17 (73.9%)
Diagnosis of PE	26 (22.6%)	6 (26.1%)
**Repeat test 4-6 weeks**	**Normal test result**	**Abnormal test result**
No PE diagnosis	69 (86.2%)	19 (65.5%)
Diagnosis of PE	11 (13.8%)	10 (34.5%)
**Repeat test 6-8 weeks**	**Normal test result**	**Abnormal test result**
No PE diagnosis	46 (85.2%)	11 (78.6%)
Diagnosis of PE	8 (14.8%)	3 (21.4%)
**Repeat test > 8 weeks**	**Normal test result**	**Abnormal test result**
No PE diagnosis	38 (88.4%)	12 (85.7%)
Diagnosis of PE	5 (11.6%)	2 (14.3%)
**Symptomatic for pre-eclampsia**		
**Repeat test < 2 weeks**	**Normal test result**	**Abnormal test result**
No PE diagnosis	29 (72.5%)	7 (70.0%)

Diagnosis of PE	11 (27.5%)	3 (30.0%)
**Repeat test 2-4 weeks**	**Normal test result**	**Abnormal test result**
No PE diagnosis	43 (81.1%)	6 (42.9%)
Diagnosis of PE	10 (18.9%)	8 (57.1%)
**Repeat test 4-6 weeks**	**Normal test result**	**Abnormal test result**
No PE diagnosis	17 (73.9%)	4 (26.7%)
Diagnosis of PE	6 (26.1%)	11 (73.3%)
**Repeat test 6-8 weeks**	**Normal test result**	**Abnormal test result**
No PE diagnosis	17 (81.0%)	6 (37.5%)
Diagnosis of PE	4 (19.1%)	10 (62.5%)
**Repeat test > 8 weeks**	**Normal test result**	**Abnormal test result**
No PE diagnosis	11 (91.7%)	3 (37.5%)
Diagnosis of PE	1 (8.3%)	5 (62.5%)

Table S7: Primary outcome and secondary perinatal outcomes, for women with a normal first test result and repeat testing at least two weeks from the initial test

Outcome	Normal first test result n=639		
	**Revealed (intervention) n=309**	**Concealed (usual care) N=330**	**Risk ratio (95% CI)**
**Primary outcome**			
Composite	50 (16.2%)	52 (15.8%)	1.03 (0.72 – 1.47)
**Components of composite:**			
1. Stillbirth	0	1 (0.3%)	
2. Early neonatal death [a]	0	0	
3. NNU admission	50 (16.2%)	51 (15.5%)	
**Status at Birth**			
Miscarriage (22-23+6 weeks' gestation)	0	0	
Late neonatal death (8-27 complete days of life)	0	0	
Preterm delivery <37 weeks)	54 (17.5%)	48 (14.6%)	1.20 (0.84 – 1.72)
Preterm delivery <34 weeks)	12 (3.9%)	9 (2.7%)	1.42 (0.61 – 3.33)
Birthweight centile			
Birthweight centile <10th	27 (8.7%)	34 (10.3%)	0.85 (0.53 – 1.38)
Birthweight <3rd centile	10 (3.2%)	5 (1.5%)	

Survival to discharge without severe morbidity	309 (100%)	327 (99.1%)	1.01 (1.00 – 1.02)

Table S8: Secondary maternal outcomes with comparisons, stratified by first test result, for women with a normal first test result and repeat testing at least two weeks from the initial test

**Outcome**	**Normal first test result n=639**		
	**Revealed (intervention) n=309**	**Concealed (usual care) n=330**	**Risk ratio (95% CI)**
Number of individuals with adverse outcomes (defined by fullPIERS consensus)	7 (2.3%)	7 (2.1%)	1.07 (0.38 – 3.01)
Number of individuals with pre-eclampsia (including those diagnosed by trial team)	60 (19.4%)	71 (21.5%)	0.90 (0.66 – 1.23)
Systolic blood pressure ≥160mmHg	99 (32.0%)	94 (28.5%)	1.12 (0.89 – 1.42
Caesarean section (versus vaginal delivery)	193 (62.5%)	178 (53.9%)	1.16 (1.01 – 1.32)

Table S9: Time to delivery with preeclampsia (median, interquartile range) stratified by first PlGF concentration (for women receiving repeat concealed PlGF-based testing only)

Category	Time to delivery (median, IQR)	Time to delivery (median, IQR)	Category	Time to delivery (median, IQR)	Category	Time to delivery (median, IQR)	Time to delivery (median, IQR)	Category	Time to delivery (median, IQR)
First test	First test	Second test	Second test stratified by first test	Second test	First test	First test	Second test	Second test stratified by first test	Second test
**PlGF >100pg/ml**	52.0 (40.0-72.0) n=35	27.0 (18.0-46.0)	PlGF ≥100 pg/ml	34.0 (20.0-53.0) n=25	**sFlt/PlGF ≤38**	49.0 (45.0-58.5) n=27	35.0 (26.0-39.5)	sFlt/PlGF ≤38	35.0 (26.0-39.5) n=20
			PlGF 12-99 pg/ml	20.0 (18.0-24.0) n=10				sFlt/PlGF >38 to <85	13.0 (7.0-31.0) n=5
			PlGF <12 pg/ml	n=0				sFlt/PlGF ≥ 85	6.5 (2.0-11.0) n=2
**PlGF 12-99 pg/ml**	24.0 (16.0-33.0) n=54	11.0 (6.0-22.0)	PlGF ≥100 pg/ml	21.0 (16.0-46.0) n=4	**sFlt/PlGF >38 to <85**	26.0 (15.0-38.0) n=24	13.0 (8.0 – 29.0)	sFlt/PlGF ≤38	36.0 (25.0-46.0) n=4
			PlGF 12-99 pg/ml	11.0 (7.0-21.0) n=37				sFlt/PlGF >38 to <85	13.5 (8.0-26.5) n=12
			PlGF <12 pg/ml	9.0 (6.0-21.0) n=13				sFlt/PlGF ≥ 85	16.5 (9.5-21.5) n=8
**PlGF <12 pg/ml**	17.0 (14.0-25.0) n=23	8.0 (1.0-13.0)	PlGF ≥100 pg/ml	n=0	**sFlt/PlGF ≥ 85**	21.0 (14.0-28.0) n=29	11.0 (6.0-20.0)	sFlt/PlGF ≤38	n=0
			PlGF 12-99 pg/ml	7.0 (3.0-14.0) n=4				sFlt/PlGF >38 to <85	n=0
			PlGF <12 pg/ml	8.0 (1.0-13.0) n=19				sFlt/PlGF ≥ 85	11.0 (6.0-16.0) n=29
	Total = 112					Total = 80			

Table S10: Time to delivery **for any reason** (median, interquartile range) stratified by first PlGF concentration (for women receiving at least one repeat test, repeat concealed PlGF-based testing only)

Category	Time to delivery (median, IQR)	Time to delivery (median, IQR)	Category	Time to delivery (median, IQR)	Category	Time to delivery (median, IQR)	Time to delivery (median, IQR)	Category	Time to delivery (median, IQR)
First test	First test	Second test	Second test stratified by first test	Second test	First test	First test	Second test	Second test stratified by first test	Second test
**PlGF >100pg/ml**	54.0 (39.0-74.0) n=180	29.5 (17.0-49.0)	PlGF ≥100 pg/ml	36.0 (20.5-51.0) n=144	**sFlt/PlGF ≤38**	50.0 (38.0-67.0) n=129	33.0 (21.0-48.0)	sFlt/PlGF ≤38	33.5 (22.0-48.0) n=112
			PlGF 12-99 pg/ml	14.5 (7.5-20.5) n=36				sFlt/PlGF >38 to <85	12.0 (6.5-27.0) n=12
			PlGF <12 pg/ml	n=0				sFlt/PlGF ≥ 85	12.0 (11.0-12.0) n=5
**PlGF 12-99 pg/ml**	26.5 (16.0-38.0) n=106	12.5 (6.0-25.0)	PlGF ≥100 pg/ml	18.0 (6.0-28.0) n=11	**sFlt/PlGF >38 to <85**	24.0 (20.0-36.0) n=36	16.0 (8.0-23.0)	sFlt/PlGF ≤38	34.0 (22.0-49.0) n=9
			PlGF 12-99 pg/ml	11.5 (6.0-27.0) n=74				sFlt/PlGF >38 to <85	16.0 (9.0-22.0) n=19
			PlGF <12 pg/ml	12.0 (7.0-20.0) n=21				sFlt/PlGF ≥ 85	16.5 (9.5-21.5) n=8
**PlGF <12 pg/ml**	19.0 (14.0-30.0) n=29	8.0 (3.0-16.0)	PlGF ≥100 pg/ml	n=0	**sFlt/PlGF ≥ 85**	21.5 (14.0-28.0) n=33	11.5 (5.5-19.0)	sFlt/PlGF ≤38	14.5 (13.0-16.0) n=2
			PlGF 12-99 pg/ml	8.0 (6.0-16.0) n=5				sFlt/PlGF >38 to <85	n=0
			PlGF <12 pg/ml	8.0 (2.0-19.0) n=24				sFlt/PlGF ≥ 85	10.0 (4.0-16.0) n=31
	Total n=315					Total n=198			

Table S11: Baseline Demographics and clinical characteristics, stratified by test type

**Baseline characteristics**	**QuidelOrtho PlGF Test n=789**		**Roche sFlt-1/PlGF ratio n=463**	
	**Revealed repeat PlGF (intervention) n=392**	**Concealed repeat PlGF (usual care) n=397**	**Revealed repeat sFlt-1/PlGF (intervention) n=233**	**Concealed repeat sFlt-1/PlGF (usual care) n=230**
**Age (years)**	33.0 (5.6)	33.0 (5.5)	30.5 (5.7)	31.7 (5.7)
**Ethnicity**	**n=389**	**n=394**	**n=232**	**n=229**
White	258 (66.3%)	259 (65.7%)	182 (78.4%)	180 (78.6%)
Black	60 (15.4%)	58 (14.7%)	19 (8.2%)	16 (7.9%)
Asian (Indian, Pakistani, Bangladeshi, Sri Lankan)	47 (12.1%)	47 (11.9%)	26 (11.2%)	27 (11.8%)
Mixed	14 (3.6%)	17 (4.3%)	3 (1.3%)	3 (1.3%)
Other (including Chinese)	10 (2.6%)	13 (3.3%)	2 (0.9%)	3 (1.3%)
Not known	3 (0.01%)	3 (0.01%)	2 (0.9%)	3 (1.3%)
**Body-mass index (kg/m^2)**	29.3 (6.9)	29.3 (7.2)	31.6 (8.3)	31.6 (7.7)
**Smoking**				
Never	315 (80.4%)	317 (80.1%)	177 (76.0%)	172 (74.8%)
Quit before pregnancy	57 (14.5%)	54 (13.6%)	31 (13.3%)	37 (16.1%)
Smoking at booking	10 (2.6%)	12 (3.0%)	12 (5.2%)	10 (4.3%)
Smoking in pregnancy	10 (2.6%)	13 (3.3%)	13 (5.6%)	11 (4.8%)
**Deprivation Quintile n (%)**	**n=345**	**n=329**	**n=208**	**n=200**
1 (most deprived)	62 (18.0%)	52 (15.8%)	110 (52.9%)	94 (47.0%)

**2**	103 (29.9%)	111 (33.7%)	28 (13.5%)	33 (16.5%)
**3**	71 (20.6%)	74 (22.5%)	28 (13.5%)	30 (15.0%)
**4**	74 (21.4%)	60 (18.2%)	26 (12.5%)	26 (13.0%)
5 (least deprived)	35 (10.1%)	32 (9.7%)	16 (7.7%)	17 (8.5%)
**Previous pregnancies with durations of ≥24 weeks n (%)**				
0	201 (51.3%)	202 (50.9%)	103 (44.2%)	101 (43.9%)
1	106 (27.0%)	105 (26.4%)	81 (34.8%)	71 (30.9%)
≥2	85 (21.7%)	90 (22.7%)	49 (21.0%)	58 (25.2%)
**Previous pre-eclampsia (of multiparous women) n (%)**	73/191 (38.2%)	73/195 (37.4%)	46/130 (35.4%)	50/129 (38.8%)
**Medical conditions**				
Pre-existing hypertension	73/392 (18.6%)	80/397 (20.2%)	40/233 (17.2%)	43/230 (18.7%)
Pre-existing renal disease	11/392 (2.8%)	17/397 (4.3%)	12/233 (5.2%)	14/230 (6.1%)
Lupus / antiphospholipid syndrome	6/392 (6.1%)	5/397 (1.3%)	2/233 (0.9%)	5/230 (2.2%)
Type 1 or type 2 diabetes	24/392 (6.1%)	25/397 (6.3%)	28/233 (12.0%)	24/230 (10.4%)
**Systolic blood pressure at booking (mmHg)**	120 (15.7)	121 (14.5)	121 (16.1)	122 (12.9)
**Diastolic blood pressure at booking (mmHg)**	75 (11.5)	76 (11.4)	73 (11.6)	75 (10.5)
**Proteinuria at booking (≥2+ on dipstick) n (%)**	5 (1.6%)	9 (2.7%)	2/126 (1.6%)	4/110 (3.6%)
**Prophylactic aspirin prescribed n (%)**	204 (52.0%)	212 (53.4%)	141 (60.5%)	149 (64.8%)
75mg aspirin	94 (46.1%)	97 (45.8%)	9 (6.4%)	10 (6.7%)
150mg aspirin	110 (53.9%)	115 (54.2%)	132 (93.6%)	139 (93.3%)
**Gestational diabetes n (%)**	75 (19.1%)	62 (15.6%)	42 (18.0%)	36 (15.7%)

**Gestation at randomisation, weeks**				
22- 27+6	48 (12.2%)	44 (11.1%)	32 (13.7%)	37 (16.1%)
28- 31+6	112 (28.6%)	106 (26.7%)	48 (20.6%)	55 (23.9%)
32- 36+6	232 (59.2%)	247 (62.2%)	153 (65.7%)	138 (60.0%)
**Gestation at randomisation**	32.0 (3.2)	32 (3.2)	33.1 (30.0-34.9)	32.9 (29.9-34.7)

Table S12: Pregnancy characteristics at first PlGF-based test, stratified by test type

**Pregnancy characteristics**	**QuidelOrtho PlGF Test n=789**		**Roche sFlt-1/PlGF ratio n=463**	
	**Revealed repeat PlGF (intervention) n=392**	**Concealed repeat PlGF (usual care) n=397**	**Revealed repeat sFlt-1/PlGF (intervention) n=233**	**Concealed repeat sFlt-1/PlGF (usual care) n=230**
**Presenting signs and symptoms (non-exclusive) n (%)**				
New-onset hypertension	186 (47.4%)	169 (42.6%)	107 (45.9%)	102 (44.3%)
Worsening of existing hypertension	76 (19.4%)	89 (22.4%)	56 (24.0%)	63 (27.4%)
Dipstick proteinuria	159 (40.6%)	175 (44.1%)	89 (38.2%)	93 (40.4%)
Neurological symptoms	36 (9.2%)	35 (8.8%)	16 (6.9%)	12 (5.2%)
Severe headache	75 (19.1%)	71 (17.9%)	55 (23.6%)	36 (15.7%)
Epigastric or right upper quadrant pain	19 (4.8%)	24 (6.0%)	17 (7.3%)	8 (3.5%)
Liver dysfunction	19 (4.8%)	18 (4.5%)	5 (2.1%)	2 (0.9%)
Acute renal insufficiency	16 (4.1%)	16 (4.0%)	16 (6.9%)	19 (8.3%)
Thrombocytopenia	4 (1.0%)	6 (1.5%)	4 (1.7%)	1 (0.4%)
Haemolysis/falling haemoglobin	5 (1.3%)	8 (2.0%)	0	1 (0.4%)
Suspected fetal growth restriction	46 (11.7%)	56 (14.1%)	31 (13.3%)	23 (10.0%)
**Highest blood pressure in 48 hours before initial test (mmHg)**				
Systolic	140 (18.3)	138 (16.7)	142 (17.5)	142 (16.2)

Diastolic	88 (13.0)	88 (12.6)	90 (13.1)	91 (11.9)
**Highest dipstick proteinuria in 48 hours before initial test n (%)**				
None	183 (49.2%)	182 (48.4%)	100 (48.8%)	87 (42.6%)
Trace	37 (9.9%)	41 (10.9%)	17 (8.3%)	27 (13.2%)
+1	90 (24.2%)	103 (27.4%)	49 (23.9%)	49 (24.0%)
≥+2	62 (16.7%)	50 (13.3%)	39 (19.0%)	41 (20.1%)
**Fetal growth abnormalities on ultrasound in two weeks prior to initial test (non-exclusive) n (%)**	**n=197**	**n=224**	**n=158**	**n=132**
None	135 (68.5%)	157 (70.1%)	123 (77.8%)	103 (78.0%)
Abdominal Circumference <10th	29 (14.7%)	25 (11.2%)	11 (7.0%)	11 (8.3%)
Estimated Fetal Weight <10th	41 (20.8%)	52 (23.2%)	27 (17.1%)	20 (15.2%)
Umbilical Artery Pulsatility Index >95th	17 (8.6%)	18 (8.0%)	8 (5.1%)	2 (1.5%)
Absent or Reduced End Diastolic Flow	7 (3.6%)	2 (0.9%)	4 (2.5%)	3 (2.3%)
Middle Cerebral Artery Pulsatility Index (centiles not available)	1.78 (0.4)	1.84 (0.4)	1.98 (0.51)	1.61 (0.55)
Amniotic Fluid Index <5th	4 (2.0%)	5 (2.2%)	0	1 (0.8%)
**Initial PlGF-based test result n (%)**				
<100 pg/ml or sFlt-1/PlGF >38	194 (49.5%)	185 (46.6%)	81 (34.8%)	76 (33.0%)
<12 pg/ml or sFlt-1/PlGF ≥85	74 (18.9%)	52 (13.1%)	39 (16.7%)	36 (15.7%)
12-99 pg/ml or sFlt-1/PlGF 38.1 – 85	120 (30.6%)	133 (33.5%)	42 (18.0%)	40 (17.4%)

≥100 pg/ml or sFlt-1/PlGF ≤38	198 (50.5%)	212 (53.4%)	152 (65.2%)	154 (67.0%)
**Initial destination n (%)**				
Home with follow up	280 (71.4%)	301 (75.8%)	196 (84.1%)	201 (87.4%)
Admitted to hospital	112 (28.6%)	96 (24.2%)	37 (15.9%)	29 (12.6%)
Other	2 (0.5%)	2 (0.5%)	1 (0.4%)	1 (0.4%)
Antenatal ward	100 (25.5%)	84 (21.2%)	34 (14.6%)	27 (11.7%)
Labour ward	6 (1.5%)	6 (1.5%)	2 (0.9%)	1 (0.4%)
Obstetric high dependency unit	2 (0.5%)	4 (1.0%)	0	0
Intrauterine transfer	2 (0.5%)	2 (0.5%)	2 (0.5%)	2 (0.5%)

Table S13: Primary outcome and secondary perinatal outcomes, stratified by test type

**Outcomes**	**QuidelOrtho PlGF Test**<br>**n=789**			**Roche sFlt-1/PlGF ratio**<br>**n=463**		
	**Revealed (intervention) n=392**	**Concealed (usual care) n=396**	**Risk ratio (95% CI)**	**Revealed (intervention) n=234**	**Concealed (usual care) n=230**	**Risk ratio (95% CI)**
**Primary outcome**						
Composite	**121 (30.9%)**	**97 (24.5%)**	**RR 1.26 (1.00-1.58) p=0.046**	74 (31.6%)	77 (33.5%)	RR 0.94 (0.73-1.23) p=0.67
**Components of composite:**						
1. Stillbirth	1 (0.3%)	2 (0.5%)	0.51 (0.05-5.58) p=0.572	1 (0.4%)	1 (0.4%)	RR 0.98 (0.06-15.62) p=0.99
2. Early neonatal death	0	0		1 (0.4%)	1 (0.4%)	RR 0.98 (0.06-15.62) p=0.99
3. NNU admission	**120 (30.6%)**	**95 (24.0%)**	**1.28 (1.01-1.61) p=0.037**	73 (31.2%)	76 (33.0%)	0.94 (0.72-1.23) p=0.67
**Status at Birth**						
Livebirth	391 (99.7%)	394 (99.5%)		233 (99.6%)	229 (99.6%)	
Miscarriage (22-23+6 weeks' gestation)	0	0		0	0	

Late neonatal death (8-27 complete days of life)	0	0		1 (0.4%)	1 (0.4%)	
**Gestational age at delivery, weeks [b]**	**36.8 (2.74)**	**37.2 (2.37)**	**-0.45 (-0.81 to -0.09) p=0.014**	36.57 (2.75)	36.85 (2.83)	-0.28 (-0.79-0.23) p=0.276
Preterm delivery <37 weeks)	147 (37.5%)	126 (31.8%)	RR 1.18 (0.97-1.43) p=0.094	95 (40.6%)	86 (37.4%)	RR 1.09 (0.86-1.36) p=0.479
**Preterm delivery <34 weeks)**	**53 (13.5%)**	**27 (6.8%)**	**RR 1.98 (1.27-3.08) p=0.002**	37 (15.8%)	28 (12.2%)	RR 1.30 (0.82-2.05) p=0.259
Birthweight centile	42.20 (31.71)	43.75 (31.51)		45.26 (32.02)	45.08 (31.98)	
Birthweight centile <10th	90 (23.0%)	71 (17.9%)	RR 1.28 (0.97-1.70) p=0.083	36 (15.5%)	44 (19.1%)	RR 0.81 (0.54-1.21) p=0.305
**Descriptive perinatal outcomes:**						
Necrotising enterocolitis (Bell's stage 2 or 3)	1 (0.3%)	2 (0.5%)		0	1 (0.4%)	
Sepsis	3 (0.8%)	2 (0.5%)		2 (0.9%)	4 (1.7%)	
Brain injury on imaging	4 (1.0%)	1 (0.3%)		0	1 (0.4%)	
Seizures	1 (0.3%)	1 (0.3%)		0	0	
Retinopathy of prematurity	3 (0.8%)	2 (0.5%)		2 (0.9%)	1 (0.4%)	
Chronic lung disease	4 (1.0%)	5 (1.3%)		3 (1.3%)	3 (1.3%)	
Umbilical artery pH	7.24 (0.08)	7.24 (0.08)		7.24 (0.07)	7.21 (0.11)	
Birthweight <3rd centile	28 (7.2%)	17 (4.3%)		14 (6.0%)	18 (7.8%)	
Survival to discharge without severe morbidity	377 (96.2%)	378 (95.5)	RR 1.01 (0.98-1.04) p=0.625	220 (94.0%)	220 (95.7%)	RR 0.98 (0.94-1.03) p=0.427

**Infant outcome**						
Discharged home	373 (95.2%)	378 (95.5%)		226 (96.6%)	216 (93.9%)	
Transferred to another hospital	17 (4.3%)	11 (2.8%)		5 (2.1%)	9 (3.9%)	
Died before discharge	0	0		2 (0.9%)	3 (1.3%)	

Table S14: Secondary maternal outcomes with comparisons, stratified by test type

**Outcomes**	**QuidelOrtho PlGF Test n=789**			**Roche sFlt-1/PlGF ratio n=463**		
	**Revealed (intervention) n=392**	**Concealed (usual care) n=396**	**Risk ratio (95% CI)**	**Revealed (intervention) n=233**	**Concealed (usual care) n=230**	**Risk ratio (95% CI)**
Number of individuals with adverse outcomes (defined by fullPIERS consensus)	10 (2.6%)	11 (2.8%)	0.90 (0.39-2.09) p=0.802	8 (3.4%)	5 (2.2%)	1.58 (0.52-4.76) p=0.412
Number of women with pre-eclampsia (including those diagnosed by trial team) (%)	163 (41.6%)	155 (39.1%)	1.06 (0.90-1.26) p=0.485	92 (39.5%)	95 (41.3%)	0.96 (0.77-1.19) p=0.69
Systolic blood pressure ≥160mmHg	152 (38.8%)	134 (33.8%)	1.15 (0.95-1.38) p=0.15	102 (43.8%)	102 (44.3%)	0.99 (0.80-1.21) p=0.902
**Caesarean section (versus vaginal delivery)**	**271 (69.1%)**	**232 (58.6%)**	**1.18 (1.06-1.31) p=0.002**	156 (67.0%)	143 (62.2%)	1.08 (0.94-1.23) p=0.282
Time to diagnosis of Pre-eclampsia (first PlGF-based test to diagnosis)	20.3 (22.2)	23.1 (23.5)	-2.77 (-7.83-2.29) p=0.282	17.0 (16.8)	21.6 (21.8)	-4.62 (-10.2-1.0) p=0.107
Time to diagnosis of Pre-eclampsia (randomisation to	15.02 (20.98)	18.05 (21.59)	-3.02 (-7.72-1.68)	10.99 (15.60)	14.41 (19.01)	-3.42 (-8.43-1.59)

diagnosis)			p=0.207			p=0.180

Figure S1.

A. Clinical management algorithm for QuidelOrtho PlGF testing

PARROT-2

Hypertension in Pregnancy NICE Guidelines (June 2019)

with additional diagnostic test(s) for the PARROT-2 trial

**Management of hypertension in pregnancy PRIOR to diagnosis of pre-eclampsia**		
	**Maternal Monitoring**	
**BP**	**140/90–159/109 mmHg**	**≥160/110 mmHg**
Offer pharmacological treatment to all women with a BP above 140/90mmg. Aim for BP ≤135/85 mmHg		
**BP Monitoring**	1-2 per week until BP ≤135/85 mmHg	Every 15–30 mins until BP <160/110 mmHg
**Proteinuria Monitoring**	1-2 times per week	Daily
**Blood Tests**	FBC, U&E and LFT at presentation and then weekly<br>***PlGF based testing on one occasion when pre-eclampsia is suspected***	
**Admission to Hospital**	Do not admit routinely	Admit
	**Assessment of Fetal Wellbeing**	
**Fetal Heart Auscultation**	Every appointment	Every appointment
**Ultrasound**	Repeat 2-4 weekly and at diagnosis of pre-eclampsia	Repeat 2 weekly and at diagnosis of pre-eclampsia
**CTG**	Only if clinically indicated	Only if clinically indicated and at diagnosis

****INTERPRETATION OF PLGF RESULT****

****LOW OR VERY LOW PLGF RESULT IS NOT AN INDICATION FOR DELIVERY IN ITSELF****

	**PlGF ≥100 NORMAL**	**PlGF 12-99 LOW**	**PlGF <12 VERY LOW**
**Interpretation**	Test negative – normal. Pre-eclampsia ruled out. Highly unlikely to need delivery due to pre-eclampsia within 14 days.	Test positive – abnormal. Pre-eclampsia not ruled out. Increased risk for preterm delivery.	Test positive - highly abnormal. Assess as pre-eclampsia. Increased risk for preterm delivery.
**What does it mean?**	98% of women who are in this green range will not need delivery for pre-eclampsia within 14 days.	A PlGF test result <100 pg/ml will correctly identify 95-96% of women with pre-eclampsia who need delivery within 14 days.	Group at highest risk of preterm delivery and fetal growth restriction. 94% of women presenting before 35 weeks with PlGF <12 pg/ml will give birth preterm.
**Median time to delivery**	< 35 weeks: 62 days<br>35-37 weeks: 16 days	< 35 weeks: 23 days<br>35-37 weeks: 9 days	< 35 weeks: 9 days<br>35-37 weeks: 4 days
**Plan**	Continue with antenatal care as clinically indicated in combination with NICE guidance above	Consider increased surveillance, with regular monitoring and fetal ultrasound if indicated.	Assess as 'pre-eclampsia' (regardless of proteinuria) with increased surveillance and fetal ultrasound as indicated.

Additional Guidance for Management:

Hypertension in pregnancy: diagnosis and management https://www.nice.org.uk/guidance/ng133

Small-for-Gestational-Age Fetus, Investigation and Management https://www.rcog.org.uk/globalassets/documents/guidelines/gtg_31.pdf

Version 1.2 2020_02_14

Figure S1.

B. Clinical management algorithm for Roche sFlt-1/PlGF testing

PARROT-2

Hypertension in Pregnancy NICE Guidelines (June 2019)

with additional diagnostic test(s) for the PARROT-2 trial

Management of hypertension in pregnancy PRIOR to diagnosis of pre-eclampsia		
**Maternal Monitoring**		
**BP**	**140/90–159/109 mmHg**	**≥160/110 mmHg**
Offer pharmacological treatment to all women with a BP above 140/90mmg. Aim for BP ≤135/85 mmHg		
**BP Monitoring**	1-2 per week until BP ≤135/85 mmHg	Every 15–30 mins until BP <160/110 mmHg
**Proteinuria Monitoring**	1-2 times per week	Daily
**Blood Tests**	FBC, U&E and LFT at presentation and then weekly<br>***sFlt-1/PlGF based testing on one occasion when pre-eclampsia is suspected***	
**Admission to Hospital**	Do not admit routinely	Admit
**Assessment of Fetal Wellbeing**		
**Fetal Heart Auscultation**	Every appointment	Every appointment
**Ultrasound**	Repeat 2-4 weekly and at diagnosis of pre-eclampsia	Repeat 2 weekly and at diagnosis of pre-eclampsia
**CTG**	Only if clinically indicated	Only if clinically indicated and at diagnosis

****INTERPRETATION OF sFlt-1/PLGF RESULT****

****High sFlt-1/PLGF RESULT IS NOT AN INDICATION FOR DELIVERY IN ITSELF****

	**sFlt-1/PlGF ≤38 NORMAL**	**sFlt-1/PlGF 39-84 HIGH**	**sFlt-1/PlGF ≥ 85 VERY HIGH**
**Interpretation**	Test negative – normal. Pre-eclampsia ruled out. Highly unlikely to develop pre-eclampsia within 7 days.	Test positive – abnormal. Pre-eclampsia not ruled out. Increased risk for preterm delivery.	Test positive - highly abnormal. Assess as pre-eclampsia. Increased risk for preterm delivery
**What does it mean?**	99% of women who are in this green range will not develop pre-eclampsia within 7 days.	sFlt-1/PlGF test >38 will correctly identify 86% of women with pre-eclampsia who need delivery within 7 days.	The red range highlights those at highest risk of preterm delivery, including delivery of an SGA infant. SFlt-1/PlGF >85 correctly identifies 93% of women with pre-eclampsia who need delivery within 3 weeks.
**Median time to delivery**	51 days	17 days	
**Plan**	Continue with usual management	Consider increased surveillance, with regular monitoring and fetal ultrasound if indicated.	Assess as 'pre-eclampsia' (regardless of proteinuria) with admission for assessment, increased surveillance and fetal ultrasound if indicated.

Additional Guidance for Management:

Hypertension in pregnancy: diagnosis and management https://www.nice.org.uk/guidance/ng133

Small-for-Gestational-Age Fetus, Investigation and Management https://www.rcog.org.uk/globalassets/documents/guidelines/gtg_31.pdf

Figure S2. Trial profile showing participant flow

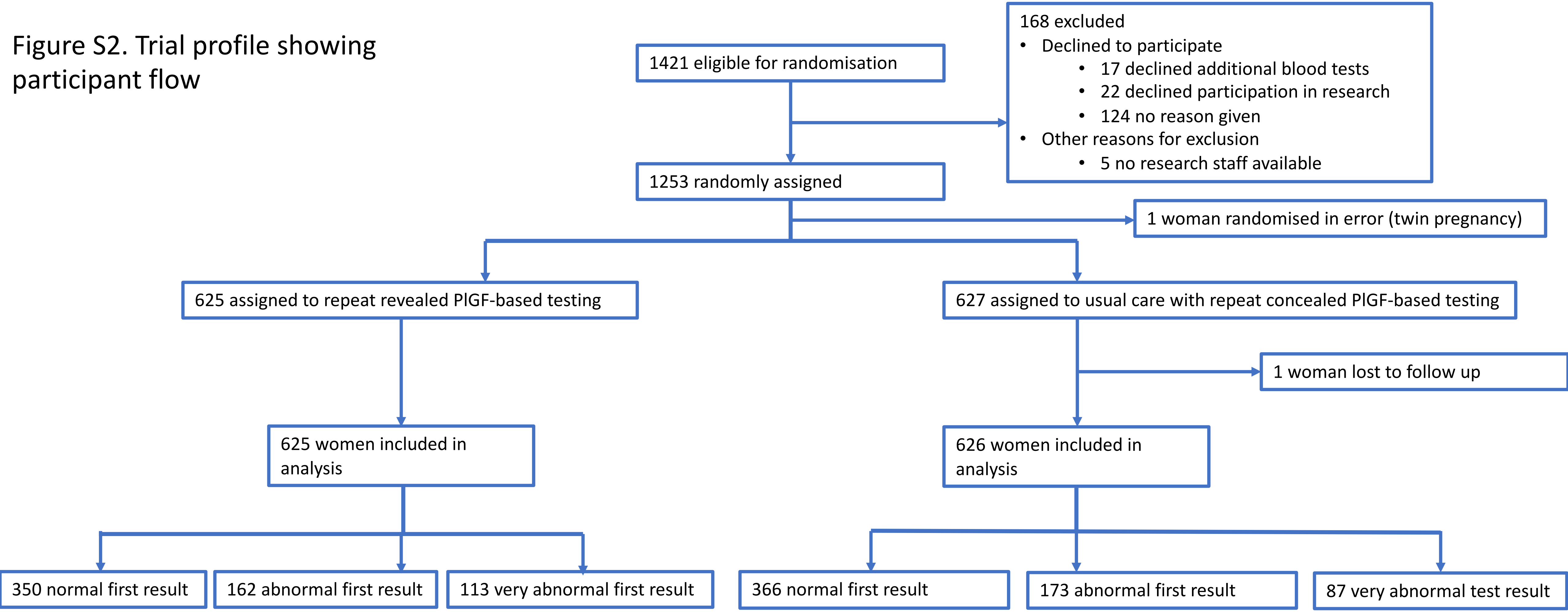

Figure S3A. Individual longitudinal measurements of PlGF (pg/ml) across gestation, in women with at least one repeat test, stratified by initial test result, and final diagnosis of pre-eclampsia (revealed and concealed groups)

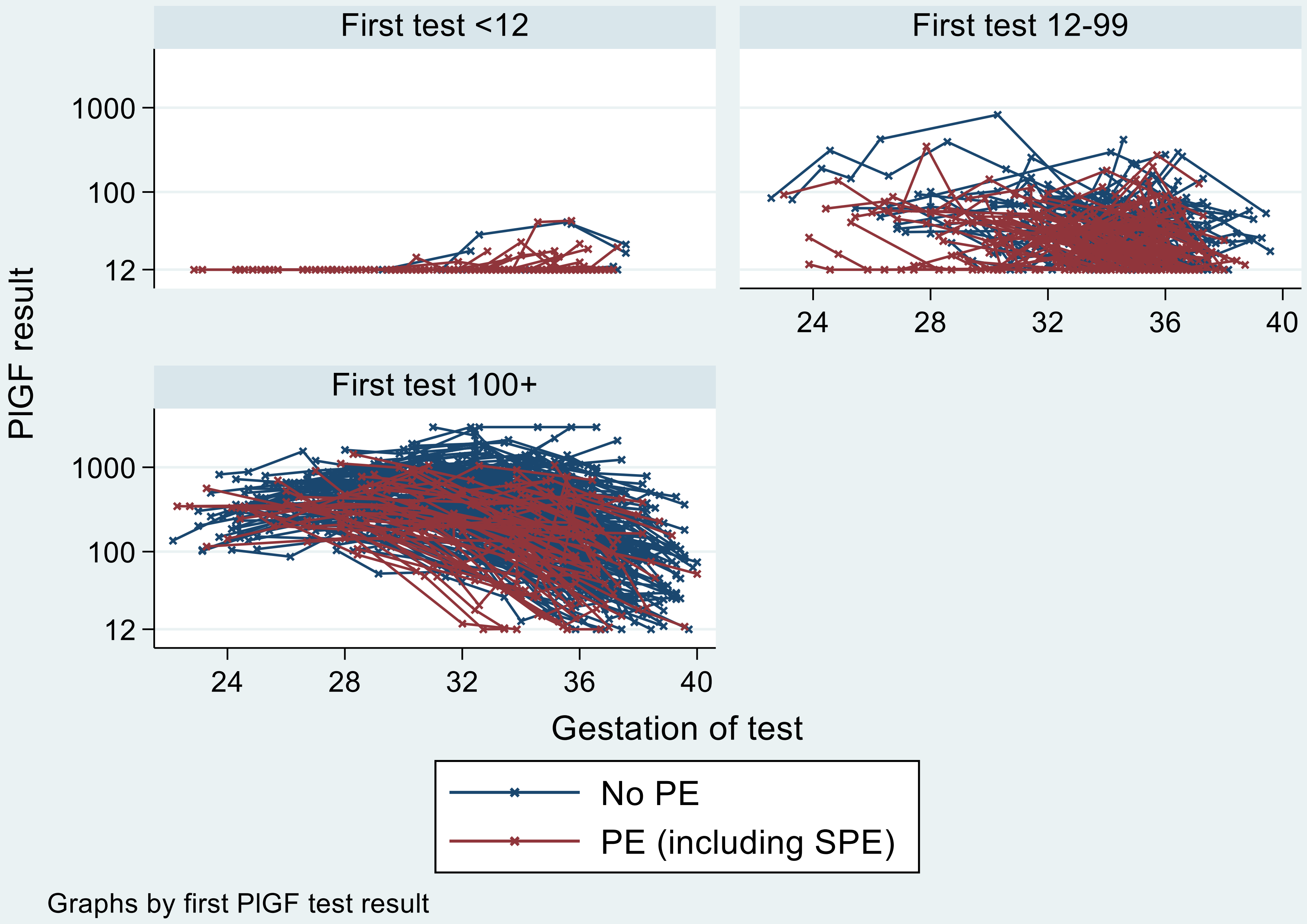

Figure S3B. Individual longitudinal measurements of sFlt-1/PlGF across gestation, in women with at least one repeat test, stratified by initial test result, and final diagnosis of pre-eclampsia (revealed and concealed groups)

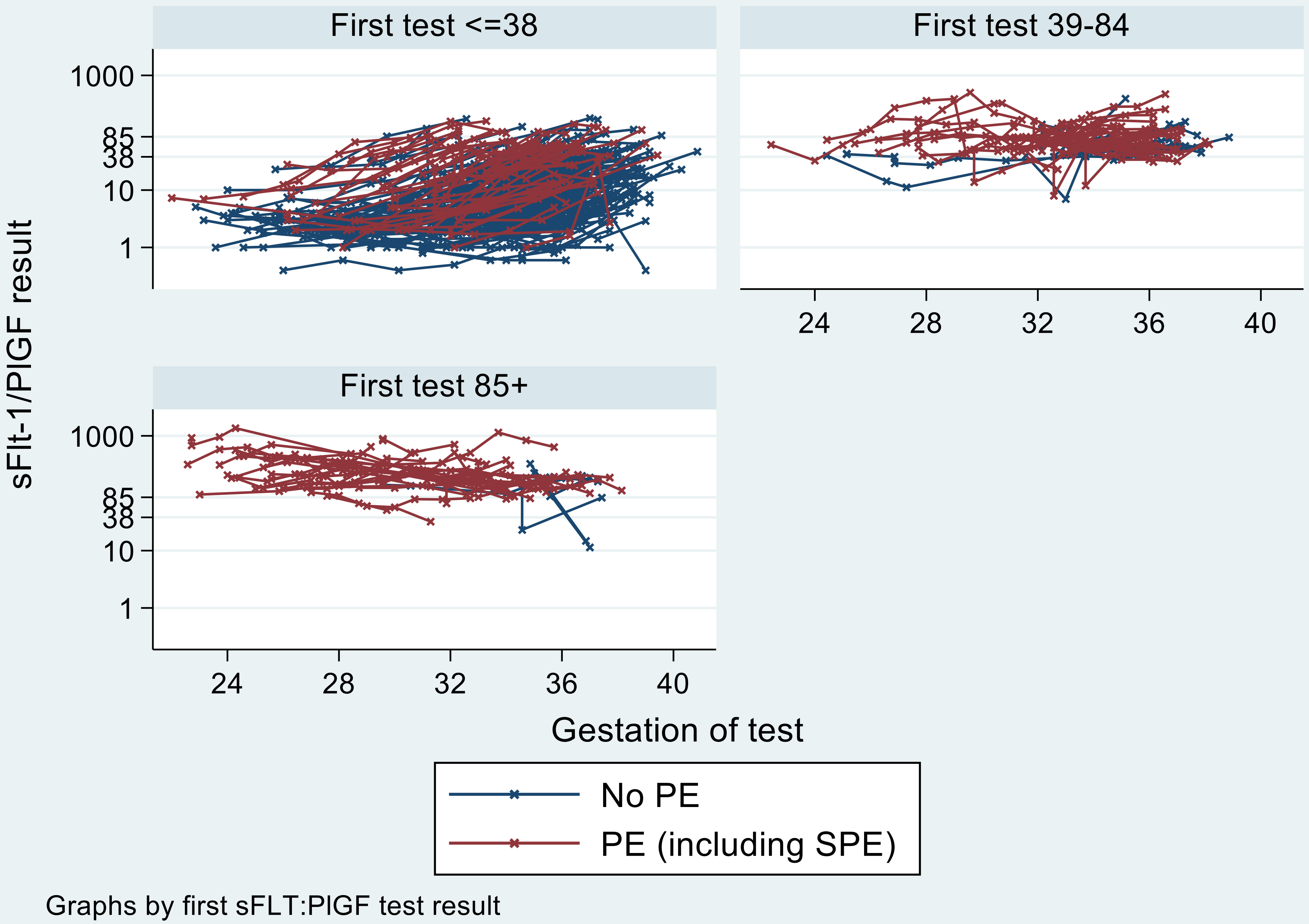

Figure S4A. Median bands of longitudinal measurements of PlGF (pg/ml) across gestation, in women with at least one repeat test, stratified by initial test result, and final diagnosis of pre-eclampsia (concealed group only)

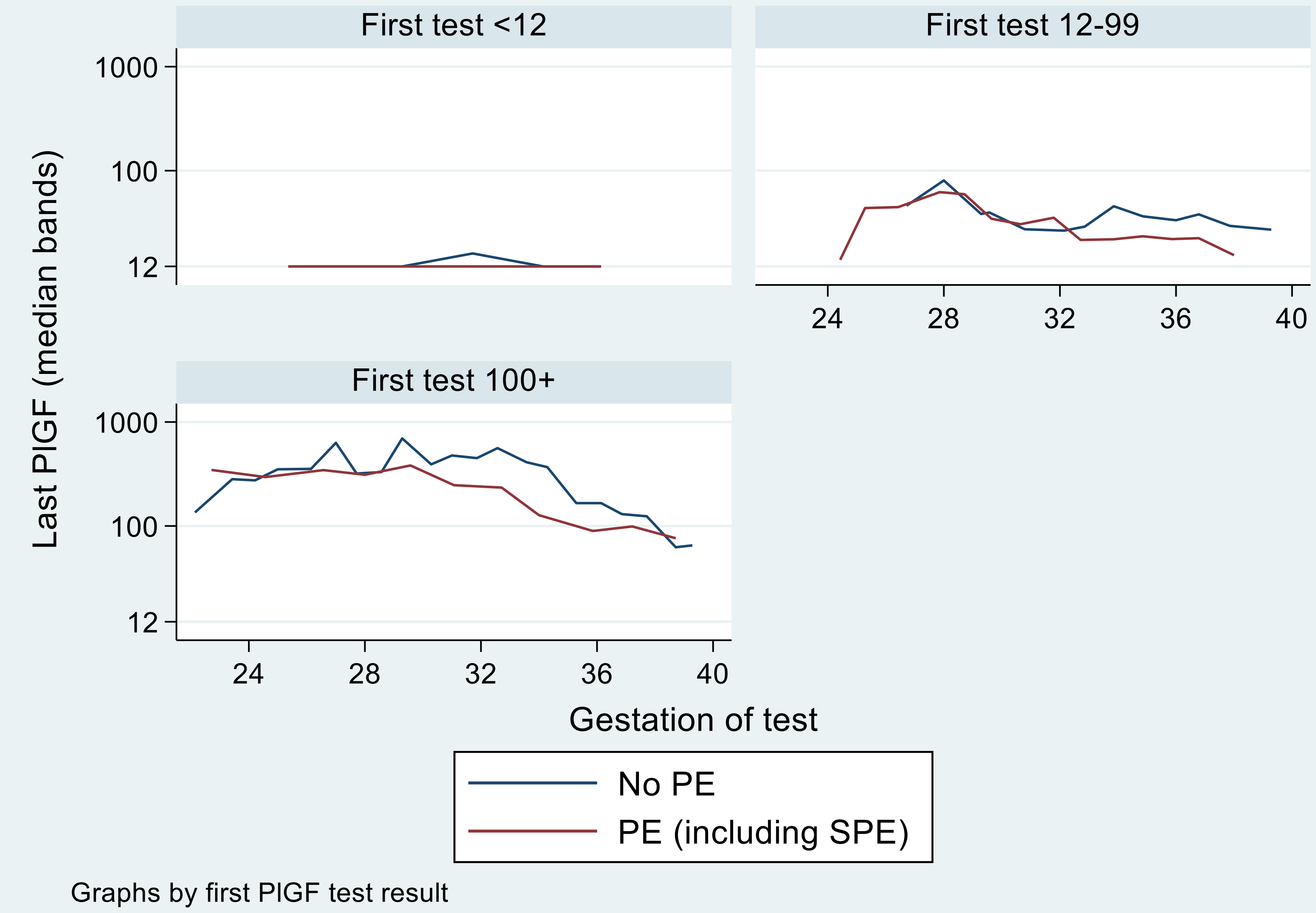

Figure S4B. Median bands of longitudinal measurements of sFlt-1/PlGF across gestation, in women with at least one repeat test, stratified by initial test result, and final diagnosis of pre-eclampsia (concealed group only)

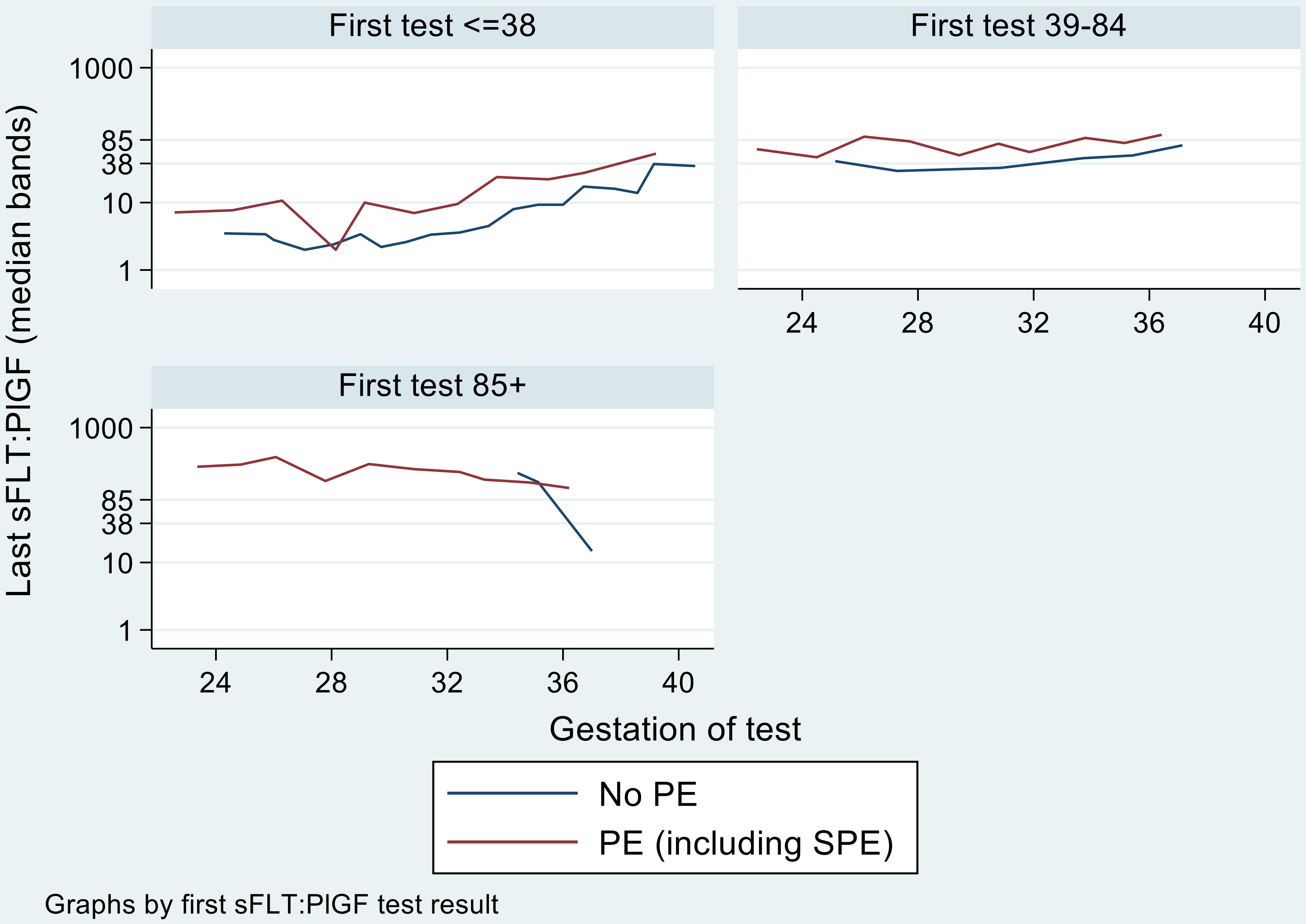

Figure S5A. Individual longitudinal measurements of PlGF (pg/ml) across gestation, in women with at least one repeat test, stratified by initial test result, and final diagnosis of pre-eclampsia (concealed group only)

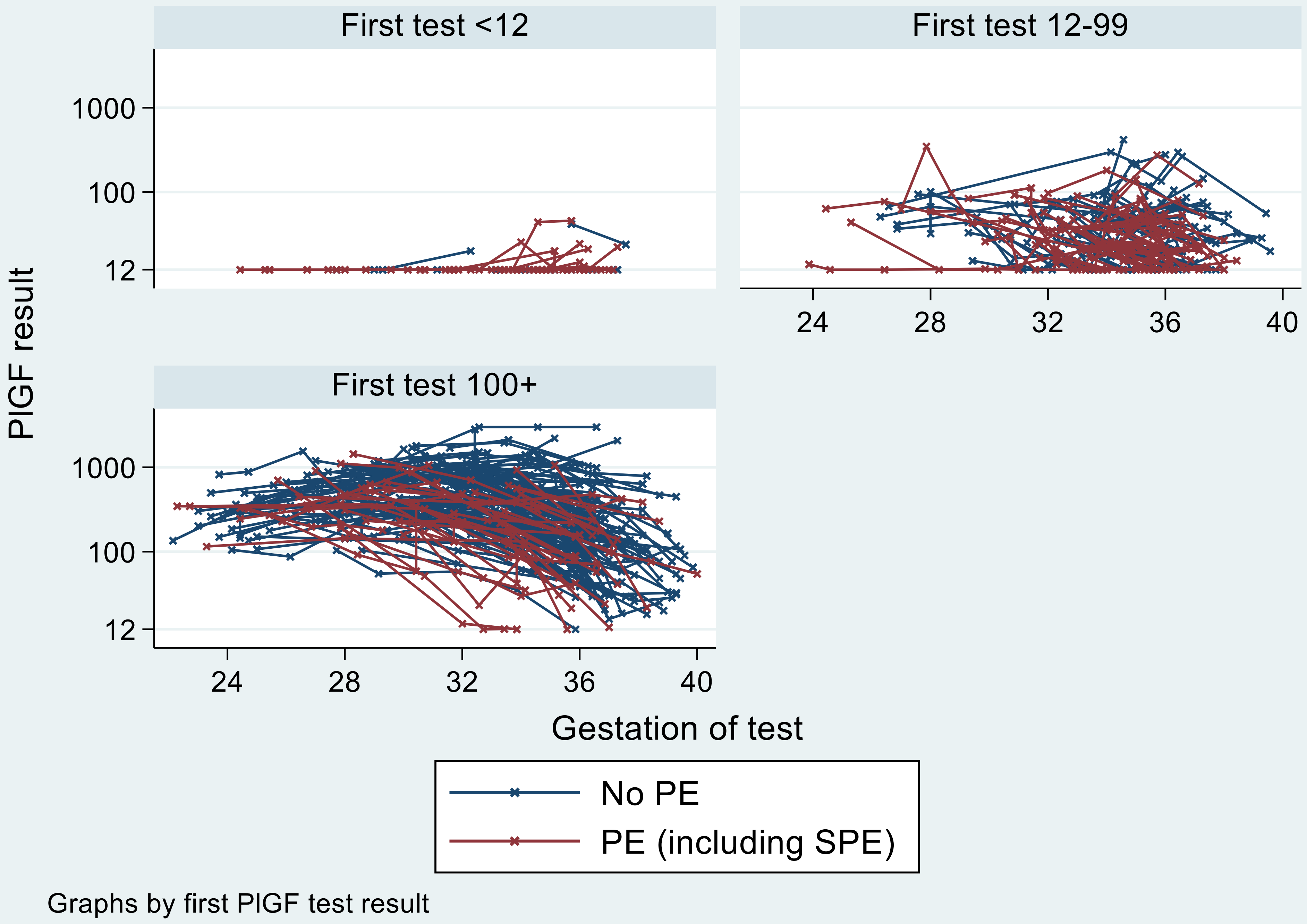

Figure S5B. Individual longitudinal measurements of sFlt-1/PlGF across gestation, in women with at least one repeat test, stratified by initial test result, and final diagnosis of pre-eclampsia (concealed group only)

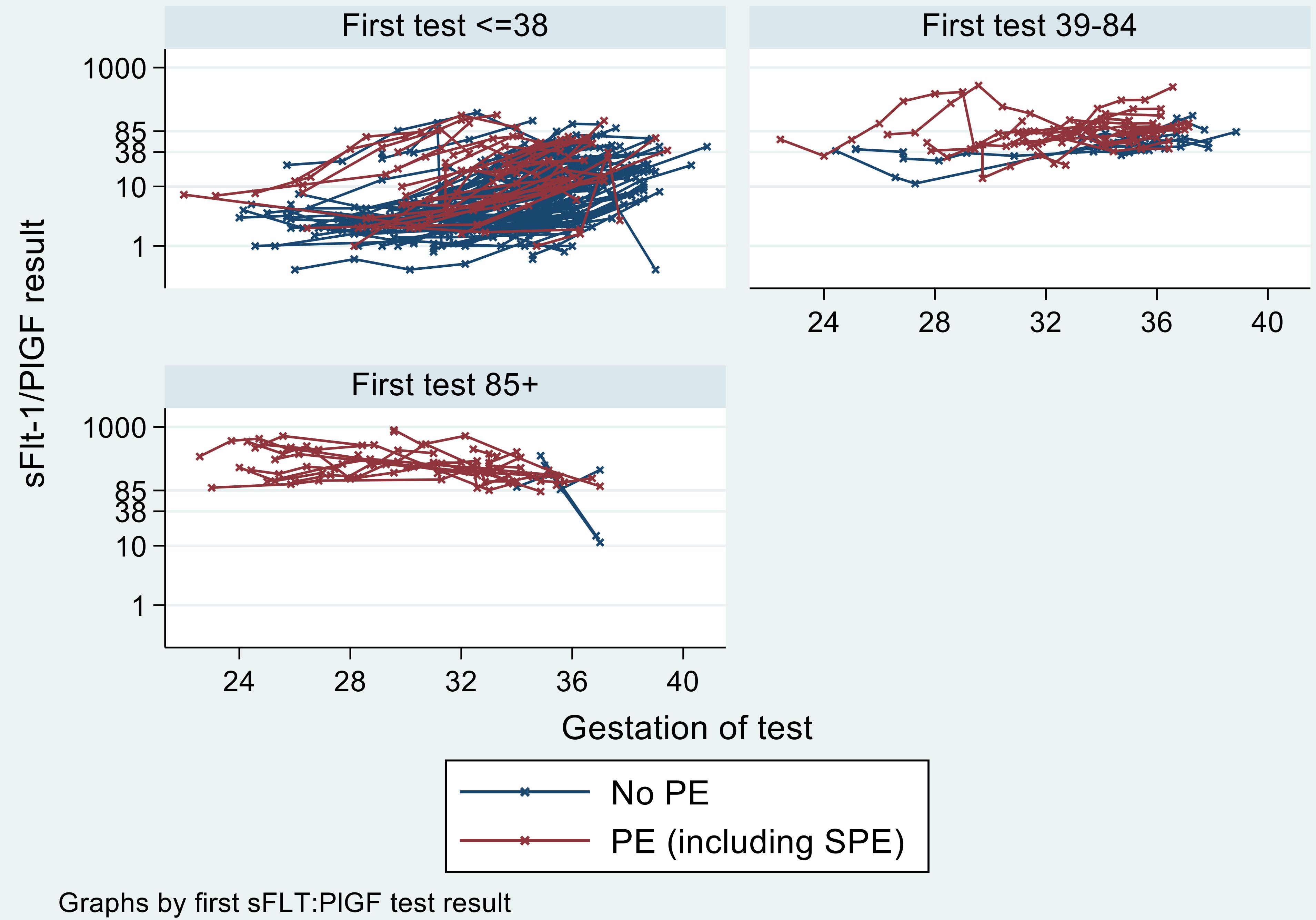

Figure S6A. Individual longitudinal measurements of PlGF (pg/ml) across gestation, in women with at least one repeat test, stratified by initial test result and fullPIERS event with final diagnosis of pre-eclampsia (revealed and concealed groups)

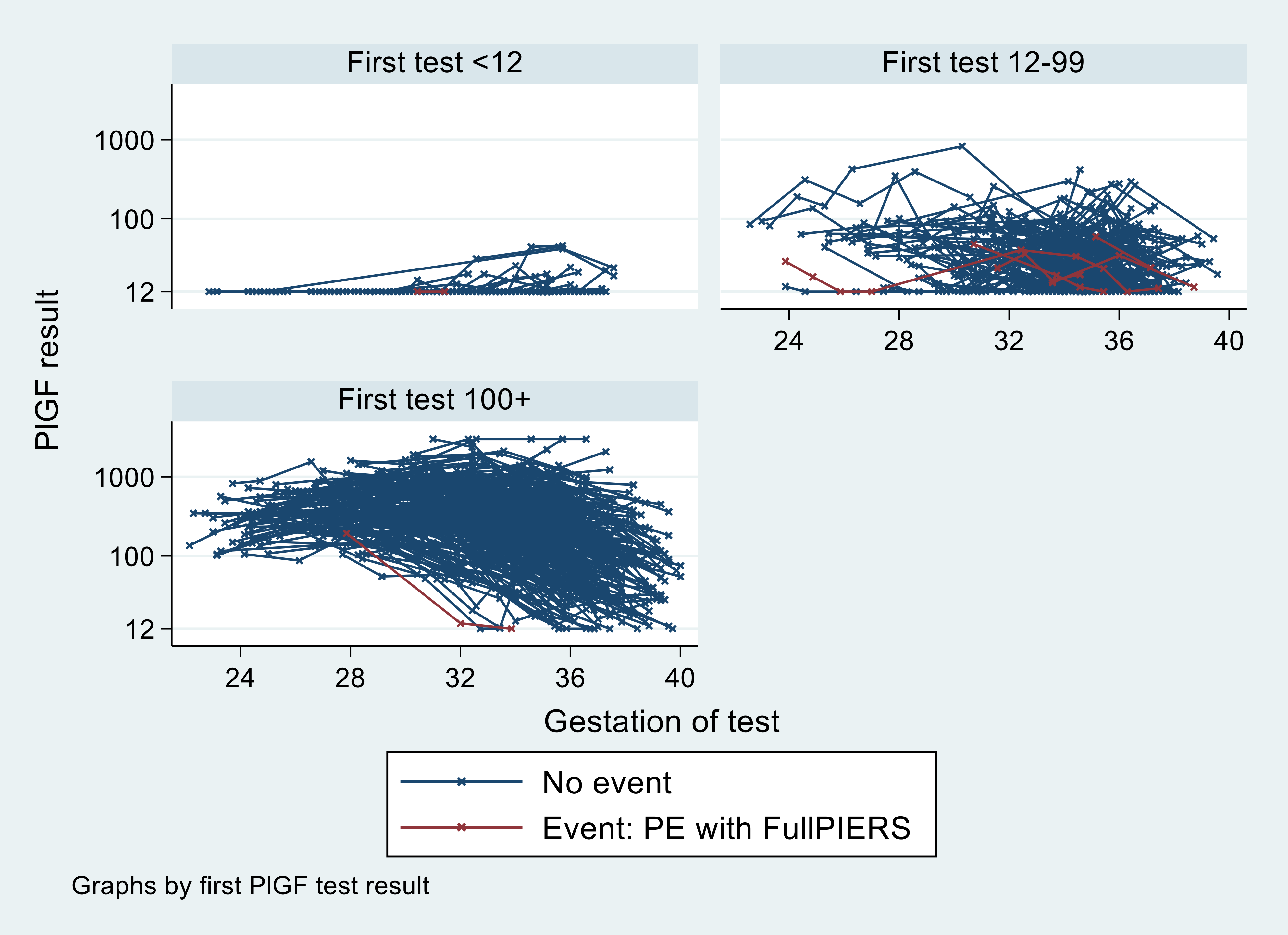

Figure S6B. Individual longitudinal measurements of sFlt-1/PlGF across gestation, in women with at least one repeat test, stratified by initial test result, and fullPIERS event with final diagnosis of pre-eclampsia (revealed and concealed groups)

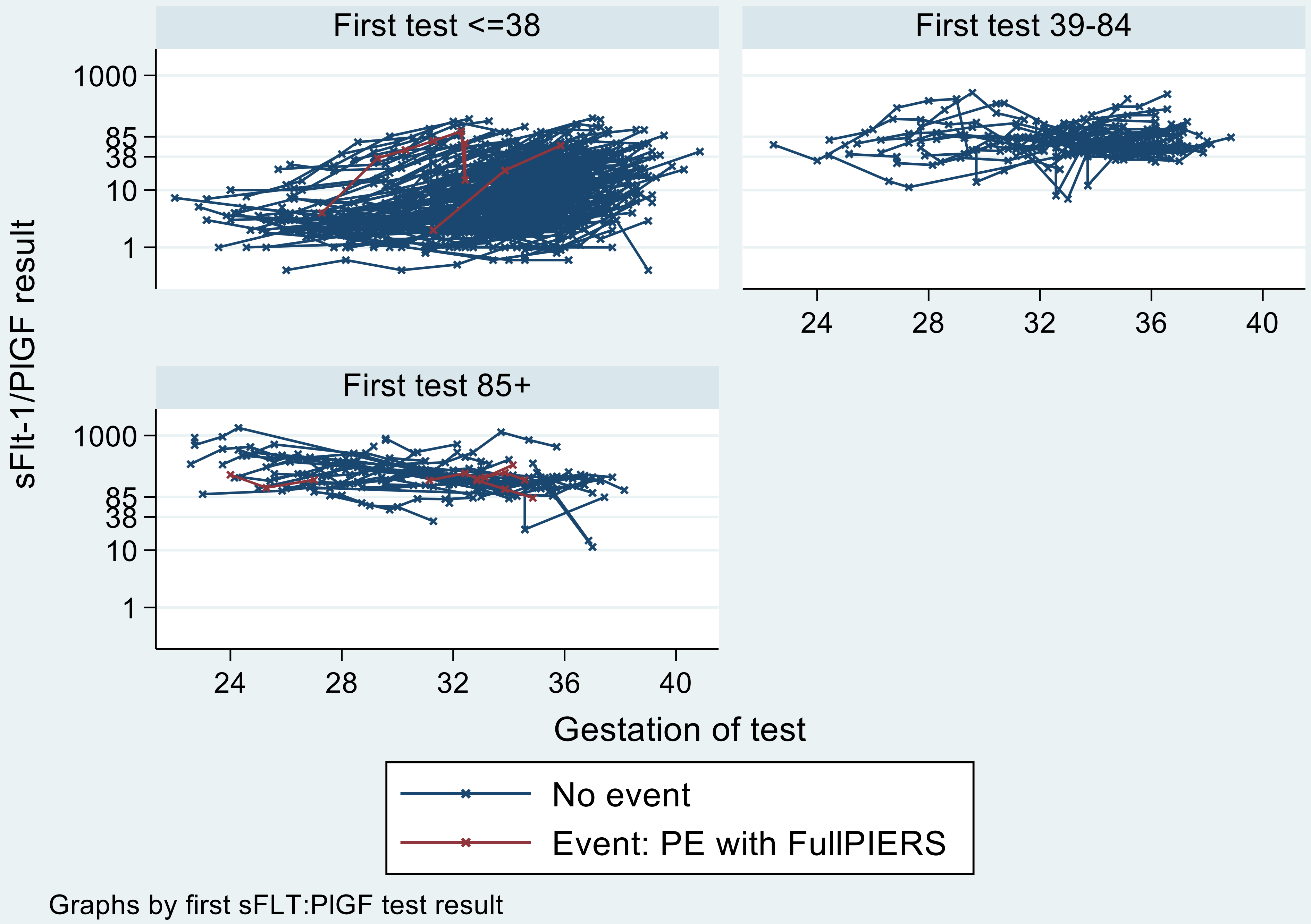

Supplement: Supplementary file 1 [file hyp-81-1561-s001.pdf]
